# Activation of protein arginine methyltransferase 1 and subsequent extension of moth lifespan is effected by the ROS/JNK/CREB signaling axis

**DOI:** 10.1016/j.jbc.2023.102950

**Published:** 2023-01-27

**Authors:** Xiao-Shuai Zhang, Wen-Sheng Li, Wei-Hua Xu

**Affiliations:** State Key Laboratory of Biocontrol, School of Life Sciences, Sun Yat-Sen University, Guangzhou, China

**Keywords:** ROS, JNK, FoxO, PRMT1, lifespan, *Helicoverpa armigera*, ChIP, chromatin immunoprecipitation, cDNA, complementary DNA, CREB, cAMP-response element binding protein, DOG, 2-deoxy-D-glucose, JNK, c-Jun N-terminal kinase, NAC, N-acetyl-L-cysteine, PQ, paraquat, PS, PRMT1-specific, PTTH, prothoracicotropic hormone, PRMT1, protein arginine methyltransferase 1, ROS, reactive oxygen species

## Abstract

Previous studies have demonstrated that high physiological levels of reactive oxygen species induce pupal diapause and extend lifespan in the moth *Helicoverpa armigera*. This has been shown to occur *via* protein arginine methyltransferase 1 (PRMT1) blockade of Akt-mediated phosphorylation of the transcription factor FoxO, after which activated FoxO promotes the initiation of diapause. However, it is unclear how PRMT1 is activated upstream of FoxO activity. Here, we show that high reactive oxygen species levels in the brains of *H. armigera* diapause-destined pupae activate the expression of c-Jun N-terminal kinase, which subsequently activates the transcription factor cAMP-response element binding protein. We show that cAMP-response element binding protein then directly binds to the PRMT1 promoter and upregulates its expression to prevent Akt-mediated FoxO phosphorylation and downstream FoxO nuclear localization. This novel finding that c-Jun N-terminal kinase promotes FoxO nuclear localization in a PRMT1-dependent manner to regulate pupal diapause reveals a complex regulatory mechanism in extending the healthspan of *H. armigera*.

Insects are the most successful animal group, and it is estimated that the total number of living insect species is over one million (1). The developmental plan of insects, which incorporates metamorphosis and diapause, must contribute to the profusion of insect species (2). Diapause confers several adaptive advantages to ensure survival in extremely harsh environments and synchronize the growth rates of populations (3) and is a reliable predicator of future seasons of adversity, such as the short days and temperatures of early autumn that accurately foretell the advent of winter. In the moth cotton bollworm, *Helicoverpa armigera*, pupal diapause is induced by incubating larvae under short-day length conditions (10 h light/day and 20 °C). Under these conditions, pupae will enter diapause 8 to 10 days after pupation and will maintain the pupal stage for over 3 months. In contrast, the larvae are incubated under long-day length conditions (14 h light/day and 20 °C); all pupae develop into adults within 3 weeks (4). Apparently, this species is an excellent model for lifespan research in a “non-aging” state, as the lifespan in the “non-aging” period is called the healthspan, which is defined as the functional and disease-free period that lengthens an organism’s lifespan (5, 6, 7).

It is well known that the insect brain perceives short-day signals and fails to produce or release development-related neurohormone prothoracicotropic hormone (PTTH) (2). Downregulation of PTTH causes another failure to stimulate the prothoracic glands to produce the growth hormone ecdysteroids needed to promote pupal–adult development (8) and finally leads to individuals entering diapause. Diapause is akin to dauer in *Caenorhabditis elegans* (9) and dormancy in animals (10), as all these phenotypes share the same characteristics: low metabolic activity, high stress resistance, and lifespan extension.

Reactive oxygen species (ROS) are generally associated with aging or age-related diseases, including Alzheimer’s disease, Parkinson’s disease, cancer, diabetes, and others (11, 12, 13). However, pioneering work in yeast, *C. elegans*, and *Drosophila melanogaster* has shown that increased ROS from chemical inhibitors or mutations that affect mitochondrial function or allotopic expression of fungal NADH dehydrogenase can lengthen rather than shorten lifespan (14, 15, 16). ROS act as secondary messengers in many cellular signaling pathways, including the insulin and c-Jun N-terminal kinase (JNK, also known as stress activated protein kinase) pathways, in response to stress-induced damage that can, in turn, induce beneficial adaptations (17, 18, 19). Activation of these protective signaling pathways caused by ROS may clarify their positive effects on lifespan.

FoxO, as a transcription factor, plays multiple roles in many cellular and physiological processes by integrating different signals from the insulin and JNK pathways (20). FoxO has been widely studied in dauer, diapause, healthspan, and longevity in *C. elegans*, insects, and mammals (21, 22, 23, 24). FoxO activity is normally indicated by its nucleocytoplasmic localization *via* its phosphorylation at Akt-recognized conserved residues, resulting in the export of FoxO protein from the nucleus to the cytoplasm (25, 26). In contrast to the negative effect of Akt, the effect of JNK on FoxO is positive. Activated JNK can promote nuclear FoxO localization, which is active FoxO, through direct binding and phosphorylation (27, 28, 29). The completely opposite effects of Akt and JNK on FoxO lead to the question of how cells control the homeostasis of FoxO activity when the two upstream kinases Akt and JNK are simultaneously activated by ROS.

Although increased ROS from chemical inhibitors, mutations, or allotopic expression can lengthen lifespan, it is still unknown whether physiological levels of ROS have the same effect. Recently, Zhang *et al*. (30) demonstrated that the ROS, p-Akt, and FoxO levels in the the brains of *H. armigera* diapause-destined pupae are significantly higher than those in their nondiapause counterparts and that p-Akt activated by ROS cannot phosphorylate FoxO, leading to its degradation because upregulation of protein arginine methyltransferase 1 (PRMT1) can increase FoxO methylation to block Akt-mediated FoxO phosphorylation; FoxO then promotes diapause initiation and extends the pupal lifespan. All diapause-destined pupae can develop into adults after the termination of diapause, and high ROS levels in the brains of diapause-destined pupae are a natural physiological state, indicating that high physiological levels of ROS are beneficial for extending lifespan in nonaging phases. Recently, Chen *et al*. (31) demonstrated that ROS promote pupal diapause in flesh fly *Sarcophaga crassipalpis*. However, it is unclear how PRMT1 in response to ROS signal is activated to regulate FoxO for inducing diapause and extending lifespan.

In this study, we show that high levels of ROS activate PRMT1 at the transcriptional level *via* the JNK/cAMP-response element binding protein (CREB)-mediated pathway, in which ROS activate JNK expression, after which JNK upregulates the activity of the transcription factor CREB. CREB directly binds to the *PRMT1* promoter and upregulates its expression. PRMT1 then promotes FoxO methylation to antagonize Akt-mediated phosphorylation of FoxO, as reported in Zhang *et al*. (30). This new finding that JNK promotes FoxO nuclear localization in a PRMT1-dependent manner to regulate pupal diapause shows a complex regulatory mechanism in extending the healthspan.

## Results

### ROS activate JNK in the brains of diapause-destined pupae

**Figure 1:**
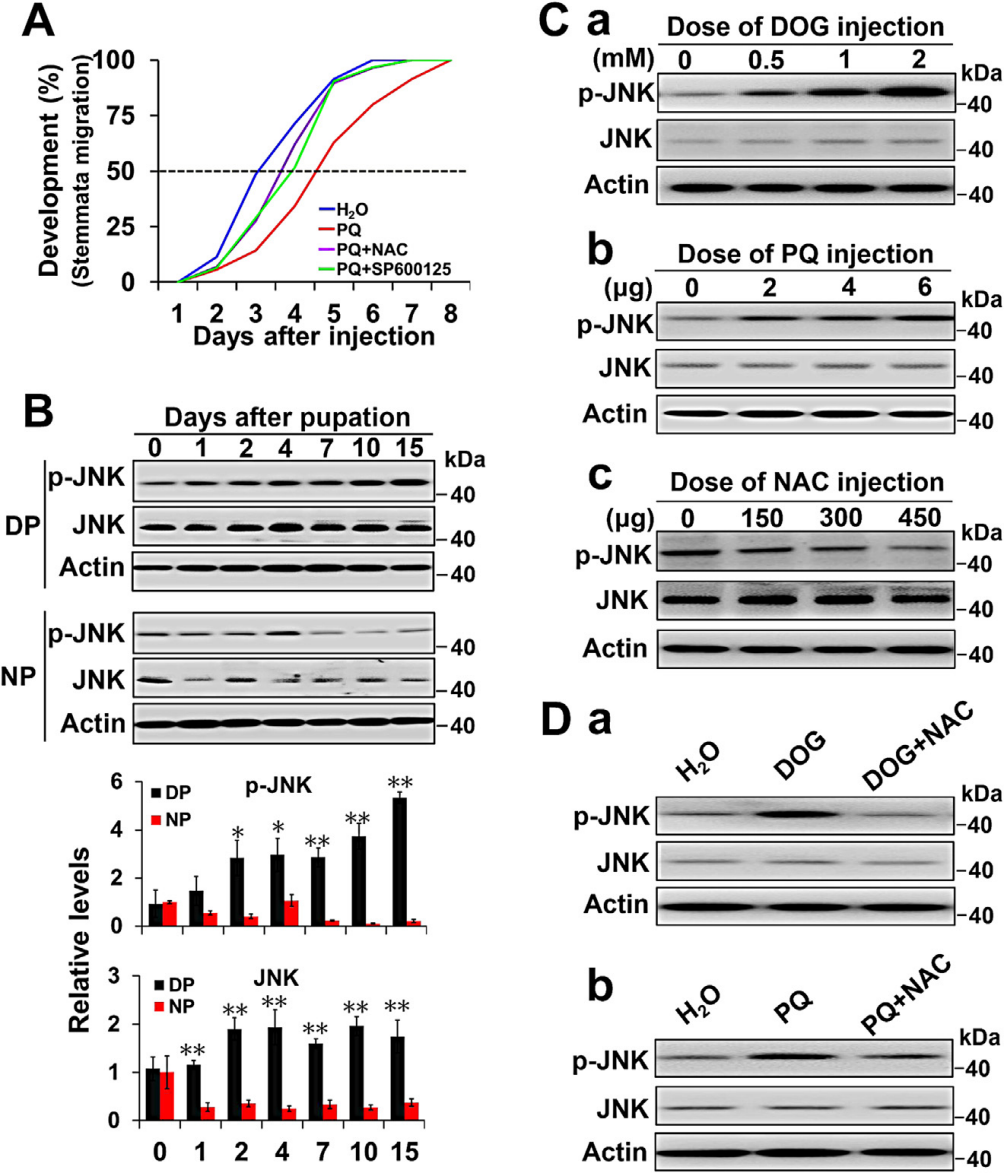
**ROS activate JNK in the brains of diapause-destined pupae.***A*, ROS delay development in *a* JNK-dependent manner. Day 1 nondiapause-destined pupae were injected with 6 μg paraquat (PQ) or with 450 μg NAC or 3 μl of 10 mM SP600125 after injection of an equal amount of PQ in 1 h. Developmental delay was determined by examining the location of the pupal stemmata on different days after injection. H_2_O, n = 35; PQ, n = 35; PQ+NAC, n = 29; PQ+SP600125, n = 30. *B*, changes in the expression of p-JNK and JNK in the pupal brain. Each point represents the mean ± S.D., *n* = 3; ∗*p* < 0.05; ∗∗*p* < 0.01. *C*, changes in p-JNK and JNK in response to the ROS generators DOG and PQ and the scavenger NAC. *a*. effects of DOG on p-JNK and JNK expression. Day 1 nondiapause-destined pupae were injected with 3 μl of the indicated dose of DOG for 48 h; *b*, effects of PQ on p-JNK and JNK expression. Day 1 nondiapause-destined pupae were injected with the indicated dose of PQ for 48 h. *c*, effects of NAC on p-JNK and JNK expression. Day 1 diapause-destined pupae were injected with the indicated dose of NAC for 48 h. The number 0 indicated that H_2_O was used as a control. Protein was extracted from brains for Western blot analysis. *D*, JNK is activated by ROS. *a*, effects of DOG and NAC on p-JNK and JNK expression in the pupal brain. Day 1 nondiapause-destined pupae were injected with 3 μl of 2 mM DOG or 3 μl of 2 mM DOG and 450 μg NAC for 48 h. *b*, effects of PQ and NAC on p-JNK and JNK expression in the pupal brain. Day 1 nondiapause-destined pupae were injected with 6 μg PQ or 6 μg PQ and 450 μg NAC for 48 h. H_2_O was used as a control. Protein was extracted from pupal brains and assessed by Western blot analysis with the corresponding antibodies. DOG, 2-deoxy-D-glucose; DP, diapause-destined pupae; JNK, c-Jun N-terminal kinase; NAC, N-acetyl-L-cysteine; NP, nondiapause-destined pupae; ROS, reactive oxygen species.

ROS can activate PRMT1 to block Akt-mediated FoxO phosphorylation, resulting in high FoxO activity to induce pupal diapause and extend lifespan in *H. armigera* (30). However, the molecular mechanisms by which ROS activate PRMT1 are unclear. Previous studies have shown that JNK, as a stress-activated protein kinase, in response to ROS can induce many beneficial adaptations (19, 28); we deduced that JNK may participate in regulating insect diapause. Thus, day 1 nondiapause-destined pupae were injected with the mitochondrial ROS generator paraquat (PQ) to increase ROS levels, and pupal development was delayed for approximately 2 days compared to the control, which was injected with solvent (Fig. 1*A*). Injection of pupae with PQ and the selective JNK inhibitor SP600125 partially rescued the developmental delay induced by PQ, similar to pupae injected with PQ and the ROS scavenger N-acetyl-L-cysteine (NAC), implying that JNK participates in the response to ROS signaling to regulate lifespan extension. Therefore, we cloned JNK complementary DNA (cDNA) from the *H. armigera* pupal brain using degenerate primers and a rapid amplification of the cDNA ends strategy. The entire Har-JNK cDNA encodes 396 amino acids (GenBank No. AAA36131) and had high identity at the amino acid level with known JNKs: 96% for *Bombyx mori*, 83% for *D. melanogaster*, and 80% for *Homo sapien*s (Fig. S1).

By Western blot analysis, both total JNK and p-JNK (the active form of JNK) proteins were significantly higher in the brains of diapause-destined pupae than in nondiapause-destined pupae, except on day 0 and day 1 for p-JNK protein and day 0 for total JNK protein (Fig. 1*B*), indicating that high JNK activity may play a role in regulating insect diapause. Day 1 nondiapause-destined pupae were injected with 2-deoxy-D-glucose (DOG, a specific inhibitor of glucose metabolism) or PQ to elevate ROS levels, and the p-JNK levels in the brain increased significantly, but injection of NAC into day 1 diapause-destined pupae caused a significant decline of p-JNK levels (Figs. 1*C* and S2*A*). Furthermore, injection of DOG and NAC or PQ and NAC into day 1 nondiapause-destined pupae caused a significant decline in p-JNK levels compared to the control, which was injected only with DOG or PQ (Figs. 1*D* and S2*B*). These results suggest that JNK can be activated by ROS in the pupal brain.

### JNK promotes FoxO nuclear localization in a PRMT1-dependent manner under oxidative stress conditions

Although PRMT1 can respond to ROS and block Akt-mediated FoxO phosphorylation to elevate FoxO activity to induce pupal diapause in *H. armigera* (30), it is unclear how PRMT1 is activated by ROS to regulate FoxO activity. Thus, we focused on the JNK-dependent function of FoxO for preventing Akt-mediated FoxO phosphorylation. Unfortunately, we found that the JNK-mediated phosphorylation sites of FoxO are not evolutionarily conserved between mammals and insects (Fig. S3). Thus, we used an anti–p-FoxO antibody against phosphorylated serine 191 (corresponding to serine 190 in *D. melanogaster* FoxO and serine 193 in *H. sapiens* FoxO4), which is an evolutionarily conserved Akt-recognized motif R-X-R-X-X-S/T (Fig. S3) (32). *H. armigera* FoxO-serine 191 is phosphorylated by Akt, and phosphorylated FoxO is then exported from the nucleus to the cytoplasm (30).

**Figure 2:**
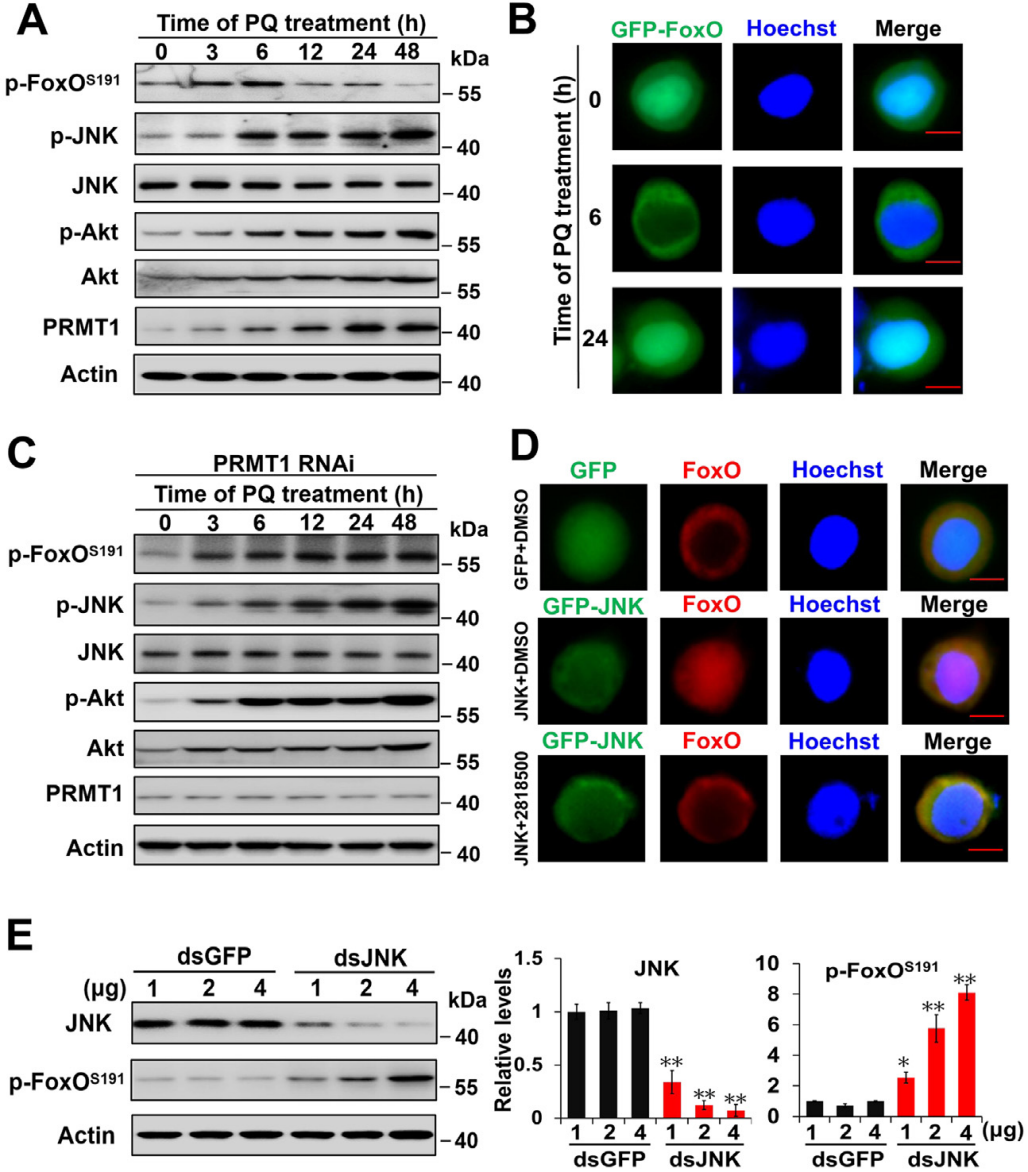
**ROS promote FoxO nuclear localization in a PRMT1-dependent manner.***A*, effects of PQ on the levels of p-FoxO^ser191^, p-JNK, JNK, p-Akt, Akt, and PRMT1. HzAm1 cells were cultured in the presence of 100 μM PQ for the indicated times. *B*, nuclear localization of FoxO in response to PQ. HzAm1 cells were transfected with the GFP-FoxO overexpression plasmid for 36 h and then treated with 100 μM PQ for the indicated times. Hoechst 33342 was used to label the nucleus. GFP-FoxO, a recombinant GFP-FoxO protein. The scale bar represents 10 μm. *C*, changes in p-FoxO^ser191^, p-Akt, Akt, p-JNK, and JNK in response to PRMT1 dsRNA treatment. HzAm1 cells were transfected with 2 μg PRMT1 dsRNA for 24 h and then treated with 100 μM PQ for the indicated times. *D*, JNK promotes FoxO nuclear localization in a PRMT1-dependent manner. HzAm1 cells were transfected with GFP or GFP-JNK for 36 h and then treated with the PRMT1 inhibitor 2818500 or DMSO as a control for 12 h. FoxO localization was detected by an anti-FoxO antibody and a *red* fluorescent secondary antibody. The scale bar represents 10 μm. *E*, changes in the p-FoxO^ser191^ levels as a result of JNK RNAi. HzAm1 cells were transfected with the indicated dose of JNK dsRNA for 48 h. GFP dsRNA was used as a control. Protein was extracted from HzAm1 cells and assessed by Western blot analysis with the corresponding antibodies. Each point represents the mean ± S.D., *n* = 3; ∗*p* < 0.05; ∗∗*p* < 0.01. JNK, c-Jun N-terminal kinase; PRMT1, protein arginine methyltransferase 1; ROS, reactive oxygen species.

HzAm1 cells were treated with PQ to imitate the long-term effect of ROS *in vivo*. The p-FoxO^ser191^ levels increased from 0 h to 6 h and then decreased from 12 h to 48 h, although the p-Akt, p-JNK, and PRMT1 levels increased gradually from 0 h to 48 h (Figs. 2*A* and S4*A*). FoxO was exported from the nucleus to the cytoplasm in response to PQ at 6 h and then reentered the nucleus at 24 h (Fig. 2*B*), consistent with the changes in the p-FoxO^ser191^ levels under oxidative stress. We speculated that FoxO is phosphorylated by Akt and promotes its export from the nucleus in 6 h and then increases JNK and PRMT1 from 12 to 48 h to antagonize Akt-mediated FoxO phosphorylation and promote FoxO nuclear localization. To test this hypothesis, cells were treated with PQ and dsRNA specific for PRMT1 to decrease PRMT1 expression; the p-FoxO^ser191^ levels increased markedly from 0 h to 48 h, accompanied by continuously increased levels of both p-Akt and p-JNK and low levels of PRMT1 (Figs. 2*C* and S4*B*), suggesting that JNK regulates FoxO nuclear localization in a PRMT1-dependent manner and that JNK cannot directly bind to and phosphorylate FoxO to prevent Akt-mediated FoxO phosphorylation when Akt is highly active. Furthermore, an immunofluorescence assay was performed in HzAm1 cells, and overexpression of JNK promoted FoxO nuclear localization compared to that in control cells overexpressing GFP, but the effect was abolished by the treatment with the PRMT1 inhibitor 2818500 (Fig. 2*D*). Moreover, silencing JNK expression in HzAm1 cells resulted in an increased level of p-FoxO^ser191^ (Fig. 2*E*). These results provide proof that JNK promotes FoxO nuclear localization in a PRMT1-dependent manner by blocking Akt-mediated FoxO phosphorylation at serine 191.

### JNK promotes PRMT1 expression by regulating its promoter activity in response to ROS

**Figure 3:**
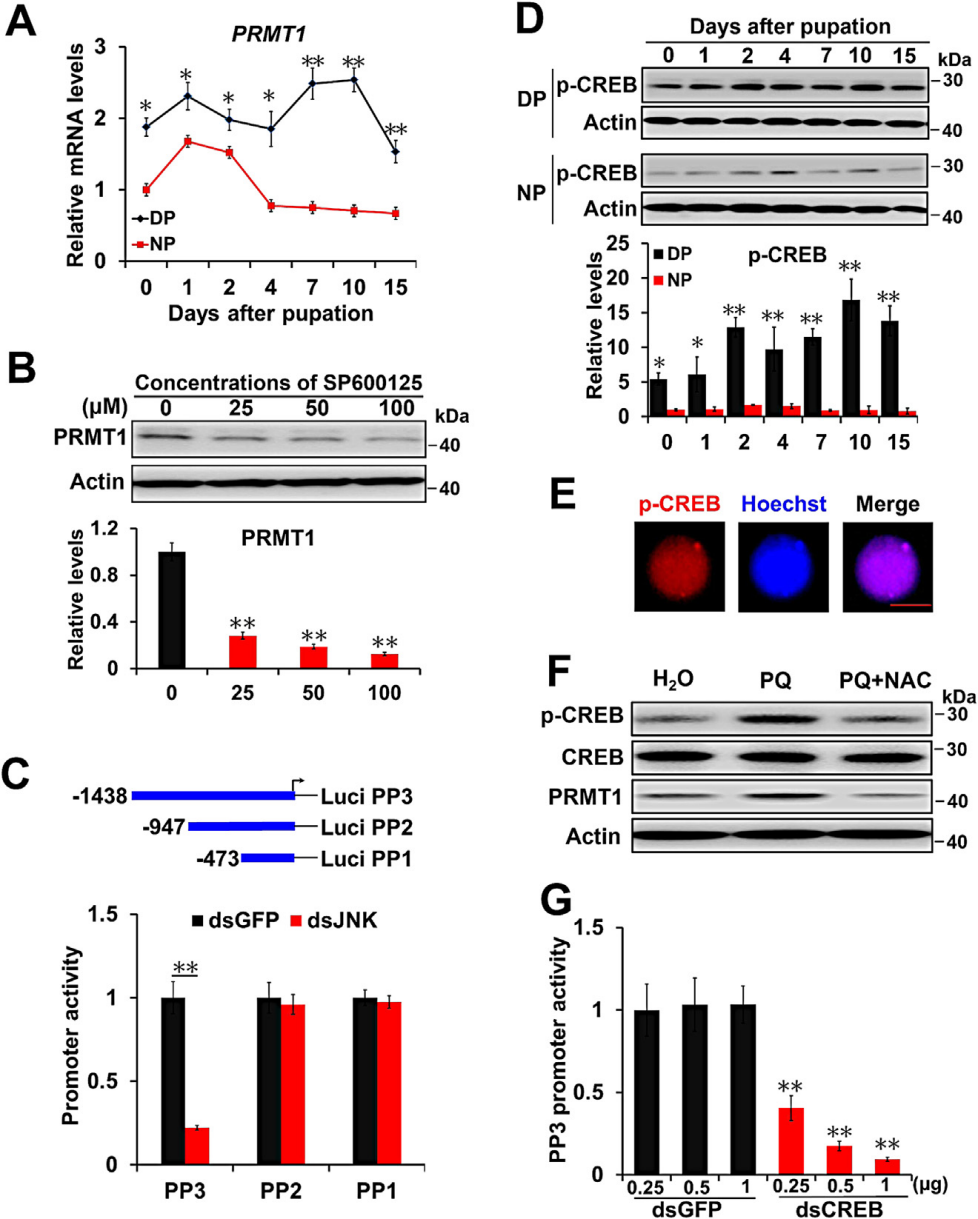
**JNK improves PRMT1 expression by regulating CREB activity.***A*, changes in *PRMT1* mRNA expression in the pupal brain. Total RNA was extracted from the brains of nondiapause- and diapause-destined pupae and used for qPCR. *H. armigera rpL32* was used as an internal standard. Each point represents the mean ± S.D., *n* = 3; ∗*p* < 0.05; ∗∗*p* < 0.01. *B*, inhibition of JNK-decreased PRMT1 protein levels. HzAm1 cells were treated with 0, 25, 50, and 100 μM SP600125, a JNK inhibitor, for 24 h. The number 0 indicated that H_2_O was used as a control. Each point represents the mean ± S.D., *n* = 3; ∗∗*p* < 0.01. *C*, truncated *PRMT1* promoter activity in response to JNK RNAi. PP1, PP2, and PP3 are *PRMT1* promoters of different lengths. GFP dsRNA was used as control. Each point represents the mean ± S.D., *n* = 4; ∗∗*p* < 0.01. *D*, changes in p-CREB levels in the pupal brain. Protein was extracted from brains for Western blot analysis. Each point represents the mean ± S.D., *n* = 3; ∗*p* < 0.05; ∗∗*p* < 0.01. *E*, nuclear localization of p-CREB. p-CREB was detected by immunostaining with an anti–p-CREB antibody. Hoechst 33342 was used to label the nucleus. The scale bar represents 10 μm. *F*, effects of PQ and NAC on p-CREB, CREB, and PRMT1 levels in the pupal brain. Day 1 nondiapause-destined pupae were injected with 6 μg PQ or with 6 μg PQ and 450 μg NAC for 48 h. H_2_O was used as a control. Protein was extracted from brains for Western blot analysis. *G*, luciferase activity assays for the *PRMT1* promoter PP3. Plasmid PP3 was cotransfected along with dsRNA specific for CREB into HzAm1 cells for 48 h, and GFP dsRNA was used as a control. Each point represents the mean ± S.D., *n* = 4; ∗∗*p* < 0.01. CREB, cAMP-response element binding protein; DP, diapause-destined pupae; JNK, c-Jun N-terminal kinase; NP, nondiapause-destined pupae; NAC, N-acetyl-L-cysteine; PRMT1, protein arginine methyltransferase 1.

Quantitative real-time PCR was performed to examine the expression of the *PRMT1* gene *in vivo*. The *PRMT1* mRNA levels in the brains of diapause-destined pupae were significantly higher than those in nondiapause-destined pupae from day 0 to day 15 (Fig. 3*A*), consistent with the PRMT1 protein levels in pupal brains, as reported in Zhang *et al*. (30). HzAm1 cells were treated with the JNK-specific inhibitor SP600125, and the PRMT1 protein levels decreased markedly (Fig. 3*B*). This result implies that JNK promotes PRMT1 expression at the transcriptional level.

To characterize the regulatory mechanism of PRMT1 gene expression, a 1438-bp fragment of the *PRMT1* promoter was cloned using the genome walking technique (33) and sequenced. Three truncations of the *PRMT1* gene promoter (PP1-3) were cloned into a pGL3-basic luciferase reporter vector and then cotransfected into HzAm1 cells with JNK or GFP dsRNA. The PP3 promoter showed decreased luciferase activity in response to JNK RNAi (Fig. 3*C*). The potential consensus sequences of the regulatory elements from −948 to −1438 of PP3 were analyzed using the JASPAR website (http://jaspar.genereg.net), and four transcription factor binding sites were predicted, including BR-C, c-Myc, CREB, and POU (Fig. S5*A*). However, the protein levels of c-Myc and POU are significantly lower in the brains of diapause-destined pupae than in those of nondiapause-destined pupae (34, 35). BR-C is required for development and metamorphosis (36). Thus, we focused on the potential CREB-binding site (5-TGAGGAAA-3), which is similar to the conserved nucleotide sequence (5-TGACGTCA-3), as CREB can respond to ROS, and its activity in brains of diapause-destined pupae is higher than that in nondiapause-destined pupae, as reported (37).

We investigated p-CREB, which is an active form of CREB, and the p-CREB levels in the brains of diapause-destined pupae were significantly higher than those in the brains of nondiapause-destined pupae (Fig. 3*D*). Furthermore, an immunofluorescence assay was performed by using an anti–p-CREB antibody, and the results showed that p-CREB was mainly localized in the nucleus (Fig. 3*E*). When PQ was injected into day 1 nondiapause-destined pupae to elevate ROS levels, the p-CREB and PRMT1 levels in brains were upregulated, but the upregulated expression was suppressed when PQ combined with NAC was injected (Figs. 3*F* and S5*B*), indicating that CREB and PRMT1 can respond to ROS. When the *PRMT1* promoter PP3 was cotransfected with CREB dsRNA into HzAm1 cells, silencing of CREB resulted in decreased *PRMT1* promoter activity compared to the control, which was treated with GFP dsRNA (Fig. 3*G*). These results indicate that CREB may be involved in regulating PRMT1 expression in response to JNK and ROS.

### CREB directly binds to the PRMT1 promoter and regulates its expression

**Figure 4:**
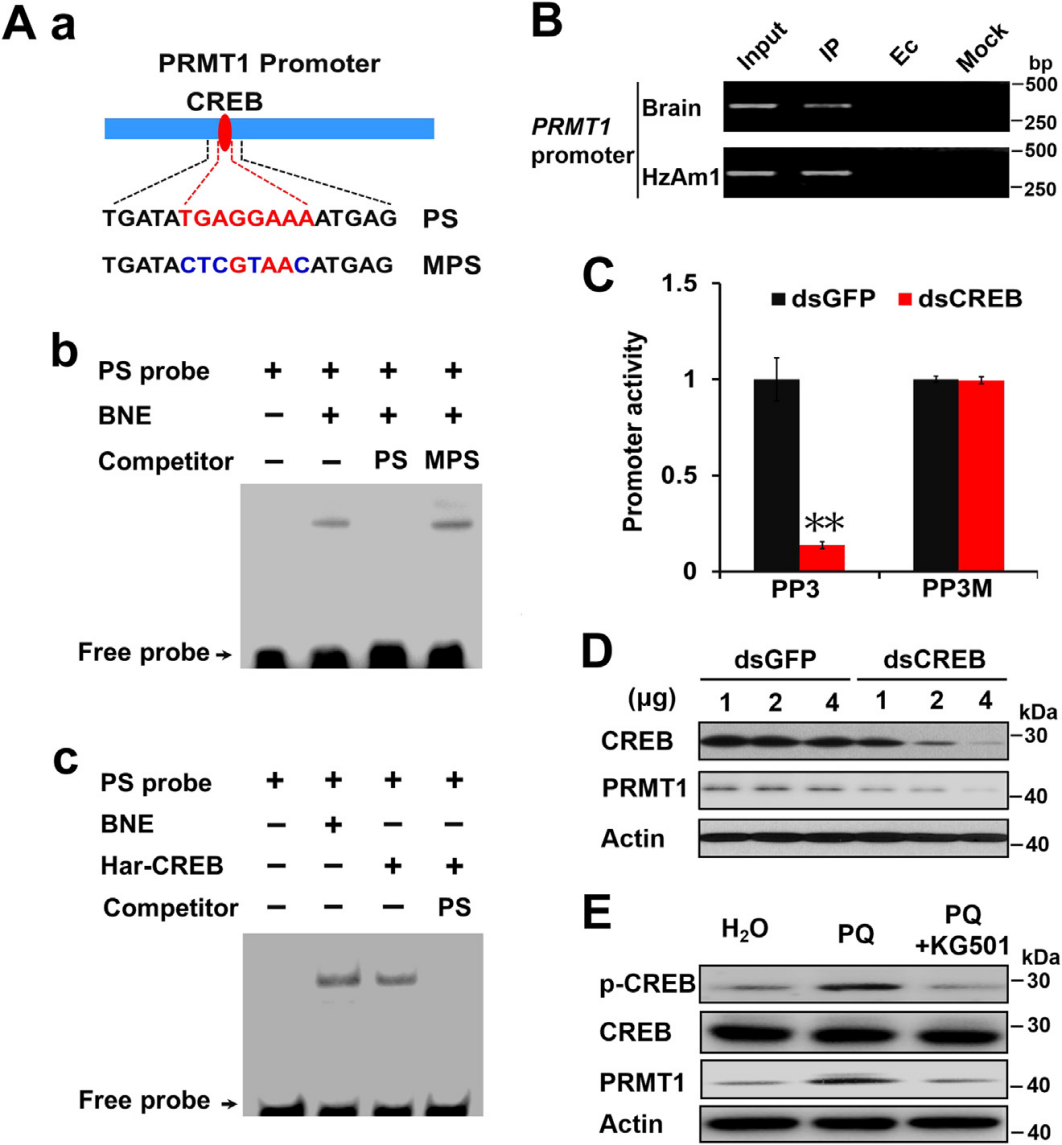
**CREB binds to the *PRMT1* promoter and regulates its expression.***A*, electrophoretic mobility shift assay (EMSA) for the CREB-binding site. *a*, schematic drawing of EMSA probes for the CREB-binding site. The *PRMT1* promoter-specific (PS) probe contains the *PRMT1* promoter’s CREB-binding region (*red*). The *PRMT1* promoter mutant-specific probe (MPS) contains a mutant CREB-binding site (*blue*). *b*, EMSA for the CREB-binding site. The probe PS was incubated with brain nuclear extract (BNE). Unlabeled probes, PS and MPS, were used as competitors. *c*, the *in vitro*-translated CREB binds PS. The probe PS was incubated with BNE or *in vitro*-translated CREB. Unlabeled probe PS was used as a competitor. *B*, ChIP assays testing CREB binding to *PRMT1* promoter in HzAm1 cells and the pupal brain. IP, cell, or pupal brain DNA immunoprecipitated using an anti-CREB antibody; Ec, empty control immunoprecipitated using preimmune serum; Mock, mock control immunoprecipitated using an anti-Actin antibody. *C*, luciferase activity assays for *PRMT1* promoter PP3 and mutant PP3 promoter PP3M. Promoter plasmids were cotransfected with CREB dsRNA into HzAm1 cells for 48 h, and luciferase activities were assessed. GFP dsRNA was used as a control. Each point represents the mean±S.D., *n* = 4; ∗∗*p* < 0.01. *D*, PRMT1 expression in response to changed CREB. HzAm1 cells were treated with the indicated dose of dsRNA specific for CREB for 48 h. GFP dsRNA was used as a control. *E*, ROS increase PRMT1 expression *via* CREB *in vivo*. Day 1 nondiapause-destined pupae were injected with 6 μg PQ or with 6 μg PQ and 0.5 μg CREB inhibitor KG501 for 48 h. H_2_O was used as a control. Protein was extracted from HzAm1 cells (figure *D*) or pupal brains (figure *E*) and assessed with the corresponding antibodies. ChIP, chromatin immunoprecipitation; CREB, cAMP-response element binding protein; MPS, mutated PRMT1-specific; PRMT1, protein arginine methyltransferase 1; ROS, reactive oxygen species.

A PRMT1-specific (PS) probe and a mutated PS probe were synthesized (Fig. 4, *A*–*a*), and PS was end-labeled by biotin. Electrophoretic mobility-shift assays (EMSAs) were performed to test whether CREB directly binds to the *cis*-element of the *PRMT1* promoter. The PS probe, containing the predicted CREB-binding site, produced a distinct shift in the pupal brain nuclear extract, and the shift could be eliminated by the 100-fold unlabeled PS probe but not by the mutated probe mutated PRMT1-specific or nonspecific probe NS as a competitor (Figs. 4, *A–b* and S6*A*). Furthermore, the *in vitro-*translated CREB protein could bind to PS, as did the control, which was incubated with PS and brain nuclear extract, and the shift could be competed with the 100-fold unlabeled PS (Fig. 4, *A*–*c*).

To clarify whether CREB binds to the *PRMT1* promoter *in vivo*, chromatin immunoprecipitation (ChIP) assays were performed in pupal brains and HzAm1 cells. The PCR product was detected when an anti–Har-CREB antibody was used, but no obvious bands were observed in the negative controls (Fig. 4*B*). Furthermore, the promoter PP3 and its mutated promoter PP3M were respectively cotransfected into HzAm1 cells with CREB or GFP dsRNA, and silencing CREB expression decreased PP3 activity but not PP3M activity (Fig. 4*C*). When HzAm1 cells were treated with dsRNA specific for CREB to downregulate CREB expression, the PRMT1 protein decreased significantly compared to the control using dsRNA specific for GFP (Figs. 4*D* and S6*B*). In addition, injection of PQ into day 1 nondiapause-destined pupae increased the p-CREB and PRMT1 levels in the brain, but injection of PQ combined with the CREB selective inhibitor KG501 failed to increase PRMT1 expression (Figs. 4*E* and S6*C*). These data show that CREB can respond to ROS signaling and that activated CREB regulates PRMT1 expression in the pupal brain.

### JNK regulates CREB activity for PRMT1 expression

**Figure 5:**
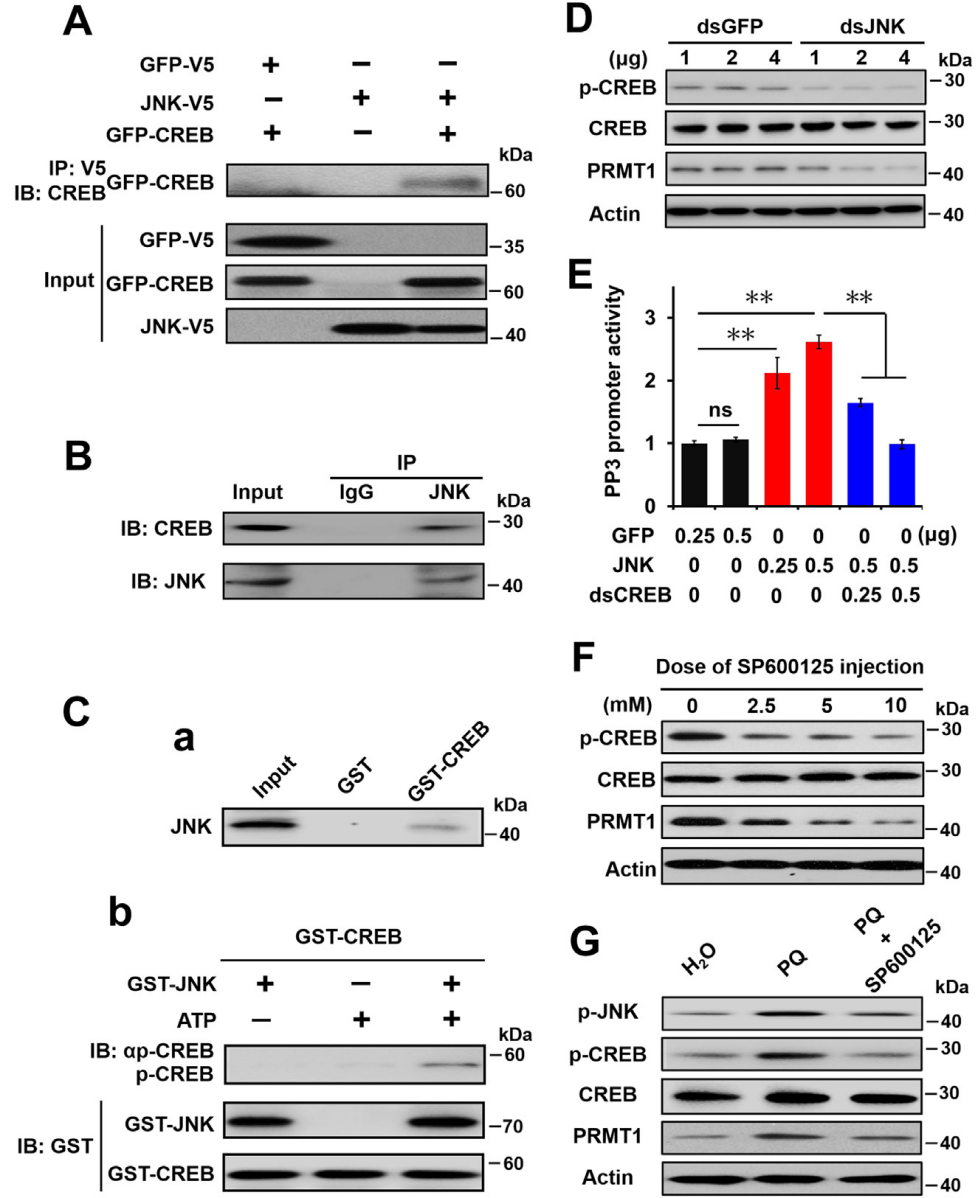
**JNK binds to and phosphorylates CREB to regulate PRMT1 expression in response to ROS.***A*, JNK physically associates with CREB by Coimmunoprecipitation in cells. HzAm1 cells were cotransfected with recombinant GFP-CREB and JNK-V5 for 48 h, and the cell extracts were immunoprecipitated with an anti-V5 antibody, followed by IB with an anti-CREB or anti-V5 antibody, respectively. *B*, JNK physically associates with CREB in the brain as determined by Coimmunoprecipitation. Brain extracts were immunoprecipitated with an anti-JNK antibody, followed by immunoblotting with an anti-CREB or anti-JNK antibody. IgG, the preimmune serum, was used as a negative control. *C*, JNK binds to and phosphorylates CREB. *a*, JNK interacts with CREB by GST pull-down assay. Brain extracts were incubated with purified GST or GST-CREB, followed by IB with an anti-JNK antibody. *b*, *In vitro* phosphorylation assay with or without GST-JNK together with GST-CREB in the presence or absence of ATP. Reaction products were analyzed by IB with an anti–p-CREB antibody. Total amounts of GST-CREB and GST-JNK were assessed by Western blot using an anti-GST antibody. *D*, p-CREB and PRMT1 expression in response to JNK dsRNA treatment. HzAm1 cells were treated with dsRNA specific for JNK for 48 h. GFP dsRNA was used as a control. *E*, JNK regulates *PRMT1* promoter activity in a CREB-dependent manner. *PRMT1* promoter PP3 was cotransfected with JNK plasmid or JNK plasmid and CREB dsRNA into HzAm1 cells for 48 h, and luciferase activity was assessed. GFP plasmid was used as a control. Each point represents the mean ± S.D., *n* = 4; ∗∗*p* < 0.01. *F*, p-CREB, CREB, and PRMT1 levels in response to the JNK inhibitor SP600125. Day 1 diapause-destined pupae were injected with 3 μl SP600125 solution for 48 h. The number 0 indicated that H_2_O was used as a control. *G*, ROS increase PRMT1 expression *via* the JNK/CREB pathway. Day 1 nondiapause-destined pupae were injected with 6 μg PQ or with 6 μg PQ and 3 μl of 10 mM SP600125 for 48 h. H_2_O was used as a control. Protein was extracted from HzAm1 cells (figure *D*) or pupal brains (figures *F* and *G*) and assessed with the corresponding antibodies. CREB, cAMP-response element binding protein; JNK, c-Jun N-terminal kinase; PRMT1, protein arginine methyltransferase 1; ROS, reactive oxygen species.

As CREB can respond to ROS signaling and regulate *PRMT1* transcription, we deduced that JNK may be involved in regulating PRMT1 expression by activating CREB. Coimmunoprecipitation was performed and showed that JNK can specifically bind to CREB in cells (Fig. 5*A*) and in the pupal brain (Fig. 5*B*). GST pull-down assays showed direct binding between JNK and CREB, and phosphorylation assays using GST-JNK and GST-CREB *in vitro* showed that JNK specifically interacts with and phosphorylates CREB (Fig. 5*C*). In addition, transfection of dsRNA specific for JNK into HzAm1 cells dramatically reduced the p-CREB and PRMT1 levels (Figs. 5*D* and S7*A*). Moreover, *PRMT1* promoter activity increased significantly when JNK plasmids were cotransfected with the *PRMT1* promoter into HzAm1 cells, but the increasing trend with overexpressed JNK was abolished by cotransfection of dsRNA specific for CREB (Fig. 5*E*). The JNK-specific inhibitor SP600125 was injected into day 1 diapause-destined pupae, after which the p-CERB and PRMT1 levels decreased, as expected, but the total CREB protein levels were unchanged (Figs. 5*F* and S7*B*). When day 1 nondiapause-destined pupae were injected with PQ and SP600125 to elevate ROS levels and decrease JNK levels, the elevated p-JNK, p-CREB, and PRMT1 induced by PQ could be resumed effectively by SP600125 (Figs. 5*G* and S7*C*). These results show that ROS promotes the expression of PRMT1 *via* the JNK/CREB pathway.

### ROS decreases p-FoxO^ser191^ through JNK-CREB-PRMT1 pathway

**Figure 6:**
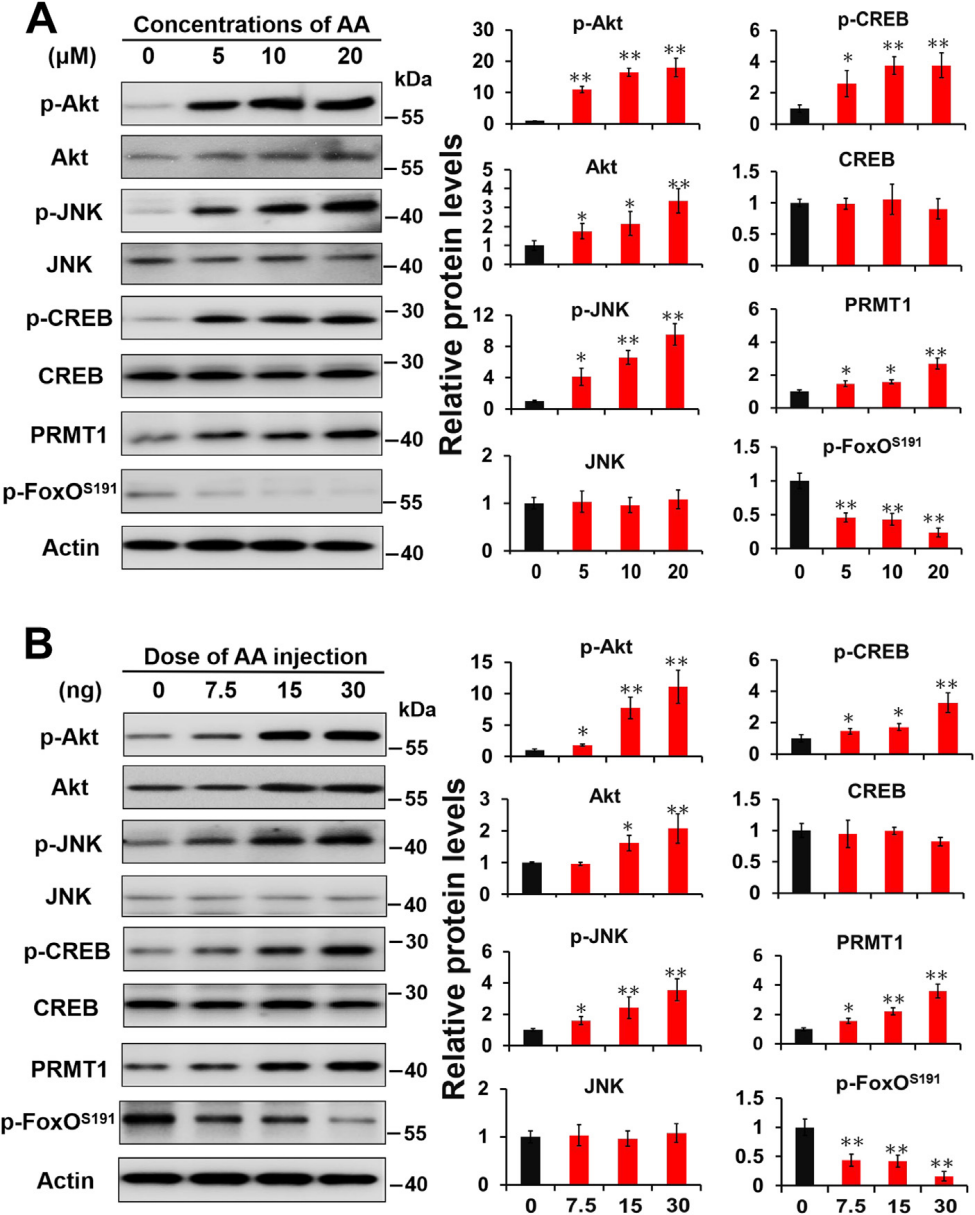
**ROS block Akt-mediated FoxO phosphorylation in a JNK-CREB-PRMT1-dependent mechanism.***A*, protein expression in response to treatment with antimycin A (AA) *in vitro*. HzAm1 cells were treated with the indicated concentration of AA for 30 min. *B*, protein expression in response to AA treatment *in vivo*. Day 1 nondiapause-destined pupae were injected with the indicated dose of AA for 4 h. The number 0 indicated that H_2_O was used as a control. Protein was extracted from HzAm1 cells or pupal brains and assessed by Western blot analysis with the corresponding antibodies. Each point represents the mean ± S.D., *n* = 3; ∗*p* < 0.05; ∗∗*p* < 0.01. CREB, cAMP-response element binding protein; JNK, c-Jun N-terminal kinase; PRMT1, protein arginine methyltransferase 1; ROS, reactive oxygen species.

As shown previously, ROS decreased the p-FoxO^ser191^ levels *via* the JNK-CREB-PRMT1 pathway. To systematically test this idea in a model, antimycin A, which accelerates mitochondrial ROS generation by inducing mitochondrial dysfunction (38, 39), was used to treat HzAm1 cells or injected into day 1 nondiapause-destined pupae. Akt-mediated FoxO phosphorylation at serine 191 increased significantly in both cells and pupal brains; however, the activated JNK-CREB-PRMT1 pathway, which contains high levels of p-JNK, p-CREB, and PRMT1, blocked Akt-mediated phosphorylation of FoxO^ser191^ and resulted in a low level of p-FoxO^ser191^ (Fig. 6, *A* and *B*). These data indicate that the ROS-JNK-CREB-PRMT1 pathway specifically blocks Akt-mediated phosphorylation of FoxO^ser191^, even though Akt is activated by ROS in diapause-destined pupae, and that activated FoxO induces insect diapause to extend lifespan.

## Discussion

Diapause confers several adaptive advantages to ensure survival in extremely harsh environments and is a complex physiological response, with many signaling pathways participating in the process, including PTTH/ecdysone (2). Reduced levels of developmental hormones lead to low metabolic activity and high ROS levels, which induce diapause. Although ROS can repress Akt-mediated FoxO phosphorylation *via* upregulation of PRMT1 (30), it remains unclear how PRMT1 is activated by ROS to regulate FoxO transcriptional activity. In this study, we provide evidence that ROS elevate FoxO activity by JNK-CREB mediated PRMT1 expression.

### JNK promotes FoxO nuclear localization in a PRMT1-dependent manner rather than through direct binding

It is well known that phosphorylation of FoxO by Akt leads to cytoplasmic localization, after which it is degraded *via* the ubiquitin-proteasome system (40), and that activated JNK can promote nuclear localization of FoxO *via* direct binding and phosphorylation in mammalian cells and *C. elegans* (27, 28, 29). Interestingly, high physiological levels of ROS cause high Akt and JNK activities and low levels of p-FoxO in the brains of *H. armigera* diapause-destined pupae to induce diapause. Zhang *et al*. (2017) demonstrated that the levels of insulin-like peptides (ILPs) are low in the brains of diapause-destined pupae and that high Akt activity responds to ROS but not to insulin-like peptides. High p-Akt levels are responsible for sensing and absorbing low levels of glucose in the blood of diapause-destined individuals by activating the glucose transporter (*Glut*), but FoxO cannot be phosphorylated by p-Akt. We speculated that JNK may participate in regulating FoxO activity to prevent Akt-mediated phosphorylation.

In this study, we found that high expression of JNK in response to ROS promotes FoxO nuclear localization by upregulating PRMT1 expression. ROS caused high expression of both Akt and JNK in 3 to 6 h, but the p-FoxO^ser191^ levels were increased by Akt for export of FoxO from the nucleus to the cytoplasm; continuously increased JNK and PRMT1 levels from 12 to 48 h caused a decrease in the p-FoxO^ser191^ levels and import of FoxO from the cytoplasm to the nucleus. To clarify whether JNK uniting PRMT1 prevents Akt-mediated FoxO phosphorylation, cells were treated with PQ and dsRNA specific for PRMT1 to decrease PRMT1 expression. The p-FoxO^ser191^ levels increased continuously from 0 h to 48 h, accompanied by high levels of both p-Akt and p-JNK. Evidently, only high JNK expression cannot block Akt-mediated FoxO phosphorylation, and JNK-mediated regulation of FoxO nuclear localization occurs in a PRMT1-dependent manner rather than JNK binding directly to and phosphorylating FoxO. Our results showed that JNK can directly bind to and phosphorylate FoxO *in vitro via* coimmunoprecipitation and phosphorylation assays (Figs. S8 and S9), as reported in mammalian cells and *C. elegans* (27, 28). We suggest that JNK uniting PRMT1 to prevent Akt-mediated FoxO phosphorylation may occur at high activities of both Akt and JNK, as in brains of diapause-destined pupae.

### JNK activates CREB to upregulate PRMT1 expression to prevent Akt-mediated FoxO phosphorylation

**Figure 7:**
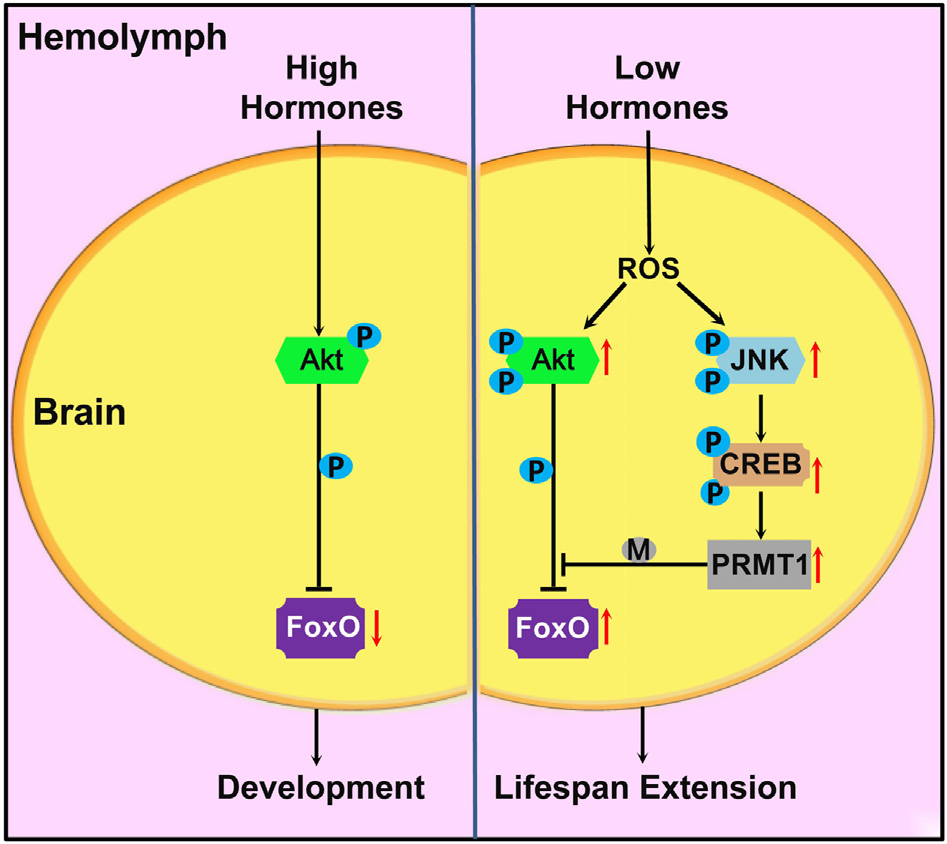
**Schematic drawing of the ROS-JNK-CREB-PRMT1-FoxO pathway for the regulation of diapause and lifespan.** High hormone levels cause Akt-mediated phosphorylation of FoxO and low FoxO activity to trigger individual development. However, low hormone levels cause high ROS levels in the brains of diapause pupae. ROS then lead to high levels of p-Akt to increase glucose intake from hemolymph, and the high activity of the JNK-CREB-PRMT1 axis antagonizes Akt-mediated FoxO phosphorylation. Activated FoxO induces insect diapause and lifespan extension. CREB, cAMP-response element binding protein; JNK, c-Jun N-terminal kinase; PRMT1, protein arginine methyltransferase 1; ROS, reactive oxygen species.

Previous studies have demonstrated that high expression of PRMT1 mediates arginine methylation of FoxO to inhibit Akt-mediated phosphorylation in *C. elegans*, *H. armigera*, and mammalian cells (30, 41, 42). However, the mechanism by which PRMT1 is upregulated remains unclear. In this study, we found that ROS-activated JNK can bind to and phosphorylate CREB and then lead to CREB translocation into the nucleus (Fig. 7). The transcription factor CREB then binds to the *PRMT1* promoter and increases its transcription. Thus, JNK-mediated FoxO methylation *via* activation of CREB and upregulation of PRMT1 expression antagonizes Akt-mediated FoxO phosphorylation and results in a low level of p-FoxO in the brain to induce pupal diapause.

In summary, previous studies on post-translational modifications of FoxO extending lifespan, including phosphorylation and methylation, mainly focused on the adult stage. However, healthspan which lengthens an organism’s lifespan in a “non-aging” state is defined recently (5, 6). Thus, this new finding that JNK promotes FoxO nuclear localization in a PRMT1-dependent manner *via* ROS-JNK-CREB-PRMT1 axis to regulate pupal diapause shows a complex regulatory mechanism in extending the healthspan.

## Experimental procedures

### Animals

*H. armigera* larvae were reared on an artificial diet at 20 ± 1 °C under a light-dark cycle of 14 h light:10 h dark (nondiapause type) or under a cycle of 10 h light:14 h dark (diapause type). All pupae of the nondiapause type developed without entering diapause, whereas approximately 95% of the diapause-type pupae entered diapause. The developmental stages were synchronized by collecting new pupae. Pupal brains were dissected in ice-cold 0.75% NaCl and stored at −80 °C until use.

### RNA extraction, DNA amplification, and developmental expression of Har-PRMT1 in the brain

Total RNA was extracted from pupal brains as reported in Chen *et al*. (34). Briefly, 1 μg of total RNA was reverse transcribed at 37 °C for 1 h using an M-MLV reverse transcription system (Takara Co, Ltd). One microliter of the reverse transcription product was added to 25 μl of the PCR mixture, and amplification was performed with specific primers designed (Supplemental Table).

The developmental expression of Har-*PRMT1* mRNA was investigated using real-time quantitative PCR. First-strand cDNA was synthesized according to the procedure described above, and PCR was performed with primers (Supplemental Table) in a Light Cycler 480 (Roche Holding AG) using SYBR Premix Ex Taq II (TaKaRa Bio Inc.). *H. armigera rpL32* was used as an internal standard.

### Polyclonal antibody generation

Partial ORFs of *Har-JNK*, *Har-FoxO*, *Har-CREB*, *Har-PRMT1*, and *Har-Actin* were amplified and cloned into the pET32a vector (Invitrogen). The recombinant proteins were expressed in BL21 (DE3) induced by IPTG at 20 °C for 9 to 12 h. The cells were lysed by ultrasonication in binding buffer (20 mM Na_3_PO_4_, 500 mM NaCl, pH 7.8), followed by centrifugation. The fractions containing the recombinant proteins were applied to an NTA-Ni^2+^-agarose cartridge (Qiagen N.V.) and eluted using elution buffer with an imidazole gradient from 50 mM to 1000 mM. The purified proteins were quantified using the Bradford method (43) and then used to generate polyclonal antibodies in rabbits, as described previously (34).

### Construction of overexpression plasmids

Full-length *FoxO*, *PRMT1*, *JNK*, and *CREB* fragments were amplified with primers containing the corresponding restriction sites listed in the Supplemental Table. The PCR products were digested and inserted into the plasmid pIZ-V5-GFP or pIZ/V5.

### Cell culture, transfection, and luciferase activity

HzAm1 cells from *Helicoverpa zea*, a close relative of *H. armigera*, were cultured at 27 °C in Grace’s insect cell culture media supplemented with 10% fetal bovine serum.

Transfections were performed using the FuGENE HD Transfection Reagent (Promega Corporation), according to the manufacturer’s instructions. Briefly, cells were suspended and plated in 96- or 24-well plates and cultured for 12 h. Plasmid DNA was mixed with the transfection reagent at a 1:3 ratio in sterile water (10 or 50 μl final volume), incubated at room temperature (RT) for 20 min and added to the wells, and the plates were gently shaken and then returned to the incubator for 48 h.

The Dual-Luciferase Reporter Assay System (Promega Corporation) was used to measure the promoter activity of *PRMT1* as previously described (4). Briefly, primers (Supplemental Table) were used to amplify *PRMT1* gene promoters of various lengths; the resulting fragments were digested with NheI and XhoI and subcloned into a similarly digested pGL3-basic vector. The pGL3-*PRMT1* vectors were transfected with or without JNK overexpression plasmids or dsRNA targeting JNK, CREB. The pRL-TK vector (Promega Corporation) was used as an internal control for determining variations in transfection efficiency. Luciferase activities were determined in triplicate in three separate experiments using a MikroWin2000 microplate luminometer (Mikrotex).

### Protein extraction and Western blot analysis

Pupal brains and HzAm1 cells were homogenized in NP40 cell lysis buffer (150 mM NaCl, 1.0% Nonidet P-40, 0.5% sodium deoxycholate, 0.1% SDS, 50 mM Tris–HCl (pH 8.0), 1 mM PMSF, 1 mM EGTA, 5 mM NaF, and 10 mM Na_3_VO_4_). The lysate was shaken in a rotary shaker for 1 h at 4 °C, followed by centrifugation for 20 min at 12,000*g* at 4 °C.

Equal amounts of protein (20 μg for p-Akt, p-JNK, JNK, CREB, p-CREB, 15 μg for p-FoxO and FoxO, 5 μg for PRMT1 and Actin) was separated on a 10% SDS-PAGE gel and transferred to a polyvinylidene fluoride membrane. The immunoreactivity was probed with antibodies against p-JNK (Cell Signaling Technology, 9251S), JNK, p-Akt (Cell Signaling Technology, 9271S), Akt and p-CREB (Cell Signaling Technology, 9198S), CREB, p-FoxO (Abcam, ab131339), FoxO, PRMT1, Actin at dilutions of 1:3000 to 1:10,000, and the secondary antibodies were applied at dilutions of 1:3000 to 1:10,000. Immobilon Western Chemiluminescent HRP Substrate (Thermo Fisher Scientific) was used for protein detection.

### Genome walking

The genomic DNA of *H. armigera* was extracted from the pupal brains according to the previous method (33), and the DNA sample was then treated with the Genome Walker Universal Kit according to the manufacturer’s protocol. The primers for primary PCR and secondary PCR were designed based on *Har-PRMT1* cDNA sequence (Supplemental Table). The samples were denatured at 94 °C for 5 min, followed by a 30–40 cycles reaction 1) primary PCR: 94 °C for 30 s; 63 °C for 30 s; 72 °C for 2 min; 2) secondary PCR: 94 °C for 30 s; 60 °C for 30 s; 72 °C for 2 min. The PCR products were separated by 1.0% agarose gel electrophoresis.

### EMSAs

Nuclear protein extracts were prepared from pupal brains using the NE-PER Nuclear and Cytoplasmic Extraction Reagents kit (Thermo Fisher Scientific) according to the manufacturer’s instructions. EMSAs were performed using the LightShift Chemiluminescent EMSA Kit (Pierce Manufacturing). Briefly, 5 μg of nuclear proteins were incubated at RT for 20 min with 20 μl containing 50 ng/μl poly(dI-dC), 2.5% glycerol, 5 mM MgCl2, 0.05% NP40, and 20 fmol of biotin end-labeled probes. The reaction mixtures were separated and then transferred onto positively charged nylon membranes. The transferred DNA was then crosslinked to the membrane by exposure to UV light for 5 min (254 nm, 1200 mJ). The membrane was then incubated with a streptavidin-horseradish peroxidase conjugate, and shifts were detected by enhanced chemiluminescence. For competition experiments, a 100-fold excess of unlabeled probe was incubated with the nuclear protein extract at RT for 20 min and then used for the above procedures.

### ChIP

ChIP assays were performed as previously described (4). Briefly, 90 pupal brains or HzAm1 cells were homogenized in 1 ml nuclear extraction buffer (10 mM Tris–HCl (pH 7.5), 0.5% Triton X-100, 3 mM CaCl_2_, 0.25 M sucrose, 1 mM PMSF, and 1 mM DTT), and formaldehyde was added to a final 1% concentration. The tubes were rotated at RT for 15 min. After sonication, the chromatin concentrations were quantified and equalized. Anti-CREB antibody and Protein G/protein A-agarose suspensions (Merck & Co, Inc, 3,418,211) were incubated with the DNA fragments for immunoprecipitations, preimmune serum served as an empty control and anti–Har-actin (an irrelevant antibody) served as a mock control. After incubation, the beads were washed four times and resuspended with 100 μl Tris–EDTA buffer and processed at 65 °C overnight to reverse the crosslinking. Finally, the DNA was purified and followed by PCR analysis.

### Immunoprecipitation, coimmunoprecipitation, and immunoblot analysis

Pupal brains and HzAm1 cells were lysed in NP-40 cell lysis buffer, and 500 μg of protein extract was used for coimmunoprecipitation. The coimmunoprecipitation systems contained 35 μl Protein G plus/Protein A-agarose suspensions (Merck Millipore, 3418211) and 1 μg V5-tag antibody (Merck Millipore, 3286106), 1 μg GFP antibody (Sangon Biotech, D110008), 1 μg JNK or FoxO antibody. The same amount of normal rabbit serum was used instead of antibodies as a negative control. Immunoblotting was performed with the corresponding antibodies, followed by incubation with Clean-blot HRP (Thermo Fisher Scientific, UB280382) 1:1000, and then the blot was detected.

### GST pull-down assay

GST Pull-down Assay was performed as described previously with some modifications (30). Briefly, GST-JNK, GST-CREB (29–250 a.a.) and GST-FoxO (147–334 a.a.) were expressed in *Escherichia coli* strain BL-21 by using the pGEX-6P vector system. *In vitro* binding assays were performed by incubating pupal brain extracts with GST-CREB (29–250 a.a.) or GST-FoxO(147–334 a.a.) immobilized on Glutathione Sepharose (GE HeathCare) in NP40 buffer. After incubation for 7 h at 4 °C, the beads were washed three times with the same buffer, and proteins were analyzed by immunoblotting.

### *In vitro* phosphorylation assay

GST-CREB and GST-FoxO (2 μg) were incubated with or without 2 μg of GST-JNK in the presence or absence of ATP (5 mM) at 37 °C for 0.5 to 1 h. The reaction products were analyzed by immunoblotting using anti–p-CREB antibody (Cell Signaling Technology, 9198S), anti–Phospho-Ser/Thr/Tyr antibody (Arigo Biolaboratories, ARG53460), and anti-GST antibody (Sango Biotech Co, Ltd, D110271).

### Statistical analysis

Statistical analyses were performed with SPSS 19.0. Paired data were analyzed using independent *t*-tests, and multiple comparisons were analyzed using one-way ANOVA. A *p* < 0.05 (∗) was considered a significant difference, and *p* < 0.01 (∗∗) indicated a highly significant difference. Error bars represent SD. All experiments were performed with three independent replicates, except for the luciferase reporter assay that was four independent replicates.

## Data availability

Data are available upon request to the corresponding author.

## Supporting information

This article contains supporting information.

## Conflict of interest

The authors declare that they have no known competing financial interests or personal relationships that could have appeared to influence the work reported in this article.
