# Supplementary material for: Activation of protein arginine methyltransferase 1 and subsequent extension of moth lifespan is effected by the ROS/JNK/CREB signaling axis
Source: J Biol Chem. 2023 Jan 27;299(3):102950. doi: 10.1016/j.jbc.2023.102950 (PMC9978625; doi:10.1016/j.jbc.2023.102950)
Supplement: Supplemental Figure [file mmc1.pdf]

Supplemental Figures

Fig. S1

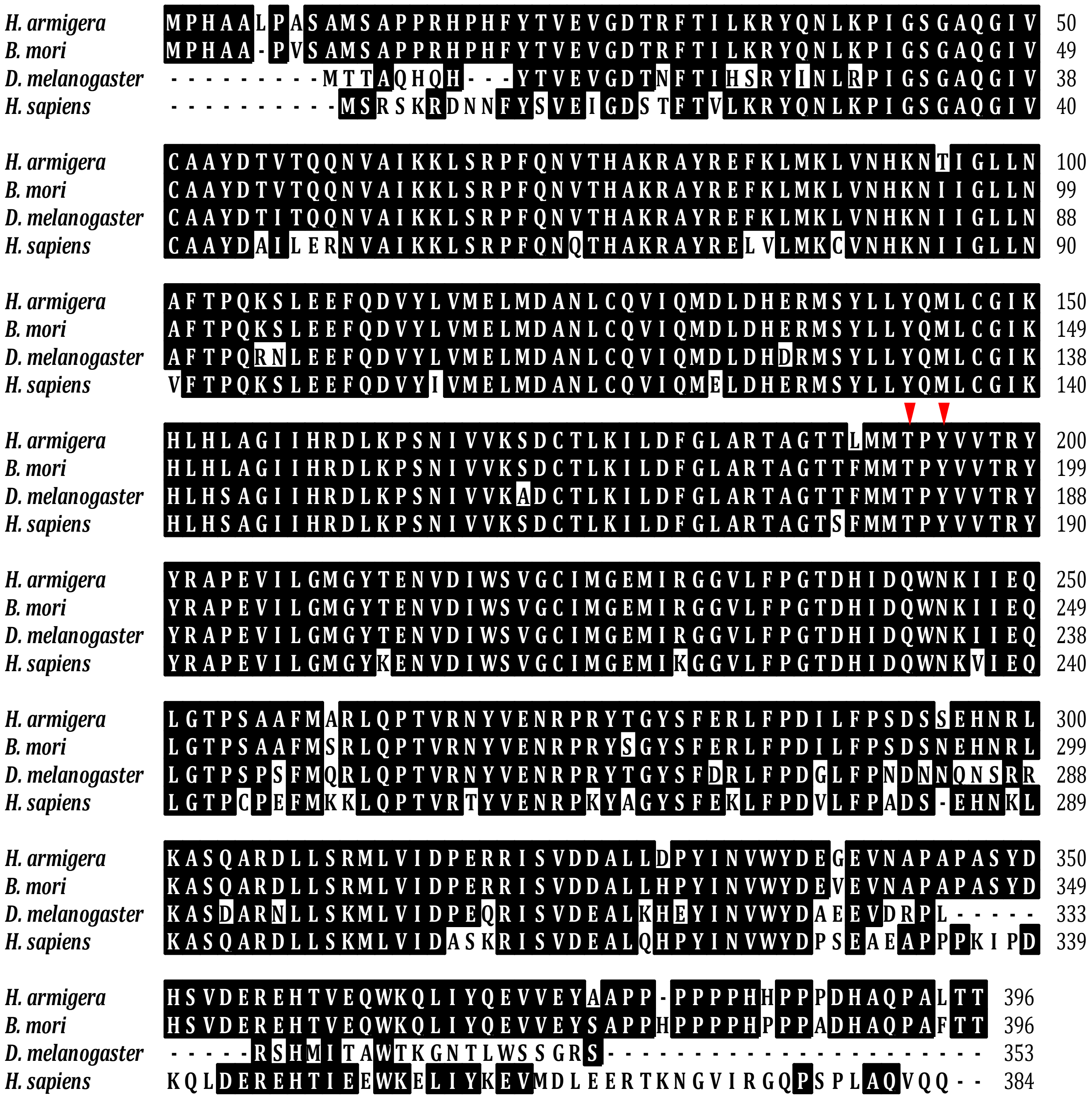

Figure S1

Homology comparison to other known JNK proteins. *H. armigera* JNK amino acid sequence has high identity with JNKs of other species: *B. mori* (96%), *D. melanogaster* (83%), and *H. sapiens* (80%). Black shading represents ≥50% sequence identity. *H. armigera*, GenBank™ number AEE81067.1; *B. mori*, NP_001103396.1; *D. melanogaster*, AAC47325.1; *H. sapiens*, NP_001310250.1. The red triangles above amino acid sequence show conserved phosphorylation sites for JNK activation.

Fig. S2

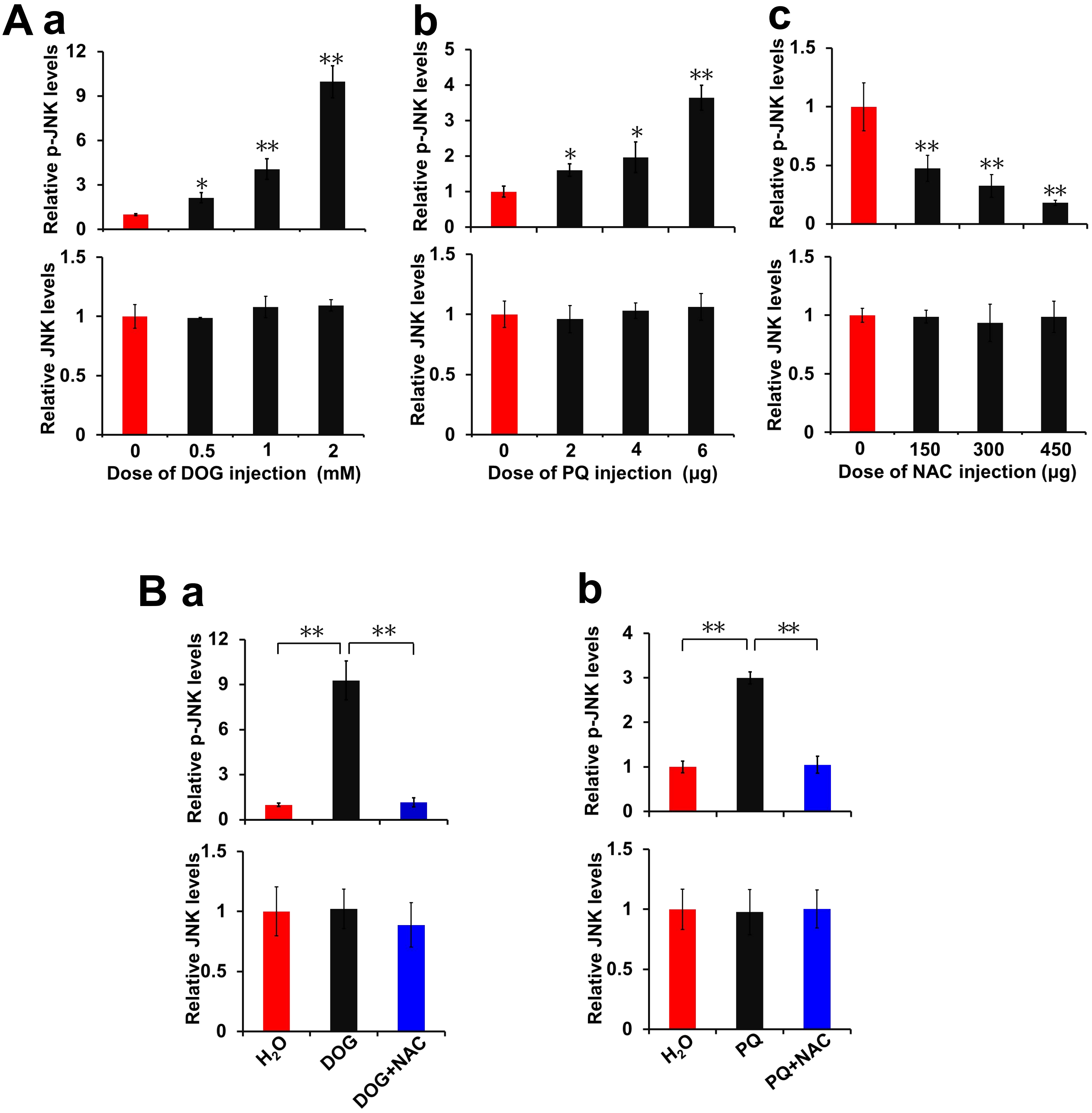

A a
Relative p-JNK levels
12
9
6
3
0
*
**
**
Relative JNK levels
1.5
1
0.5
0
0.5
1
2
Dose of DOG injection (mM)
b
Relative p-JNK levels
5
4
3
2
1
0
*
*
**
Relative JNK levels
1.5
1
0.5
0
2
4
6
Dose of PQ injection (μg)
c
Relative p-JNK levels
1.5
1
0.5
0
**
**
**
Relative JNK levels
1.5
1
0.5
0
150
300
450
Dose of NAC injection (μg)
B a
Relative p-JNK levels
12
9
6
3
0
**
**
Relative JNK levels
1.5
1
0.5
0
H2O
DOG
DOG+NAC
b
Relative p-JNK levels
4
3
2
1
0
**
**
Relative JNK levels
1.5
1
0.5
0
H2O
PQ
PQ+NAC

Figure S2

Quantification of p-JNK and JNK from Figures 1C and 1D. Western blot bands from figures 1C (A) and 1D (B) were quantified using Image software (Gel-Pro Analyzer) and normalized to the levels of Actin. Each point represents the mean±S.D., n=3; *, P<0.05; **, P<0.01.

Fig. S3

```
H.sapiens        -----------------------MRIQPQKAAAIIDLDPDFEPQSRPRSCTWPLPRPEIA  37
H.armigera       MSIRGSGGYQSPWSSQGGHSELDGTLELEPLG--ELTEVGFEPQTRARSNTWPLPRPDNY  58
D.melanogaster   ----MMDGYAQEWPR---LTHTDNGLAMDQLGGDLPLDVGFEPQTRARSNTWPCPRPENF  53

H.sapiens        NQPSEPPEVEPDLGEKVHTEGRSEPILLPSRLSEPAGGPQPGILGAVTGPRKGGSRRNAW  97
H.armigera       VEAADDTGSKKNSNQNLSG------------------APPLPAVG----TKKNSSRRNAW  96
D.melanogaster   VEPTDELDSTKASNQQLAP------------------GDSQQAIQNANAAKKNSSRRNAW  95

H.sapiens        GNQSYAEFISQAIESAPEKRLTLAQIYEWMVRTVPYFKDKGDSNSSAGWKNSIRHNLSLH  157
H.armigera       GNLSYADLITQASTSARDNRLTLSQIYEWMIQNVPYFKDKGDSNSSAGWKNSIRHNLSLH  156
D.melanogaster   GNLSYADLITHAIGSATDKRLTLSQIYEWMVQNVPYFKDKGDSNSSAGWKNSIRHNLSLH  155

H.sapiens        SKFIKVHNEATGKSSWWMLNPEGGKSGKAPRRRAASMDSSSKLLRGRSKAPKKKPSVLPA  217
H.armigera       NRFMRVQNEGTGKSSWWMINP-DAKPGKSVRRRAASMETS-KFEKRRGRVKKKAEILRTG  214
D.melanogaster   NRFMRVQNEGTGKSSWWMLNP-EAKPGKSVRRRAASMETS-RYEKRRGRAKKRVEALRQA  213

H.sapiens        P-----PEGATPTSPVGHFAKWSGSPCSRNREEADMWTTFRPRSSSNASSVSTRLSPLRP  272
H.armigera       ---ATADATPSPGSSVSESLDMFPDSPMHS--SFQLSPDFRPRVSSNASSCG-RLSPIPS  268
D.melanogaster   GVVGLNDATPSPSSSVSEGLDHFPESPLHSGGGFQLSPDFRQRASSNASSCG-RLSPIR-  271

H.sapiens        ESEVLAEEIPASVSSYAGGVPPTLNEGLELLDGLNLTSSHSLLSRSGLSGFSLQHPGVTG  332
H.armigera       MITTEHDWGPEYTDYTSANDYSQTDFGQDELAGS-LADSMKLAGTDPFLNTYVPTTSSSS  327
D.melanogaster   AQDLEPDWG-FPVDYQNTTMTQAHAQALEELTGT-MADELTLCNQQQQGFSAASGLPSQP  329

H.sapiens        PLHTYSSSLFSPAEG---------------------PLSAGEGCFSSSQALEALLTSDTP  371
H.armigera       SGGSYRYSPYGGCPRHPHG-------GCACSSLYTHPTHPAHPTHPHQHALDHFVRP--P  378
D.melanogaster   PPPPYQPPQHQQAQQQQQQQSPYALNGPASGYNTLQPQSQCLLHRSLNCSCMHNARDGLS  389

H.sapiens        PPPADVLMTQVDPILSQAPTLLLLGGLPSSSKLATGVGLCPKPLEAR------GPSSLVP  425
H.armigera       PPADPADIMRTVPFTENNQTQMVTTSDAALMN----GGMMVQTGAMG------PTTVMGR  428
D.melanogaster   PNSVTTTMSPAYPNSEPSSDSLNTYSNVVLDGPADTAALMVQQQQQQQQQQQLSASLEGQ  449

H.sapiens        TLSMIAPPPVMASAPIPKALGTPVLTPPTEAASQDRMPQDLDLDMYMEN-----------  474
H.armigera       IMGALN--TGLAEDLNIETLEHG-FDCNVDEVIKHELSMEGTLDFNFP------------  473
D.melanogaster   CLEVLNNEAQPIDEFNLENFPVGNLECNVEELLQQEMSYGGLLDINIPLATVNTNLVNSS  509

H.sapiens        ---------------------------------------LECDMDNIISDLMDEGEGLDF  495
H.armigera       -------------------------------------QQHSAMAAEAESQFAAPAPPVPT  496
D.melanogaster   SGPLSISNISNLSNISSNSGSSLSLNQLQAQLQQQQQQQAQQQQQAQQQQQQHQQHQQQ  569

H.sapiens        NFEPDP--------------------------------------  501
H.armigera       TLSGGNG------------------PRAPYS------VAPSWVH  516
D.melanogaster   LLLNNNNNSSSSLELATQTATTNLNARVQYSQPSVVTSPPSWVH  613
```

Figure S3

Homology comparison to other known FoxO proteins. *H. armigera* FoxO amino acid sequence has high identity with FoxOs of other species: *D. melanogaster* (66%), and *H. sapiens* (53%). Black shading represents ≥50% sequence identity. *D. melanogaster*, GenBank^{TM} number NP_996204.1; *H. sapie*ns, CAA63819.1. The red triangles above amino acid sequence show conserved phosphorylation sites for FoxO inactivation, the red lines show high conserved Akt phosphorylate motifs (RXRXXS). The purple triangles above amino acid sequence show phosphorylation sites for *H. sapie*ns FoxO4 by JNK in mammals.

Fig. S4

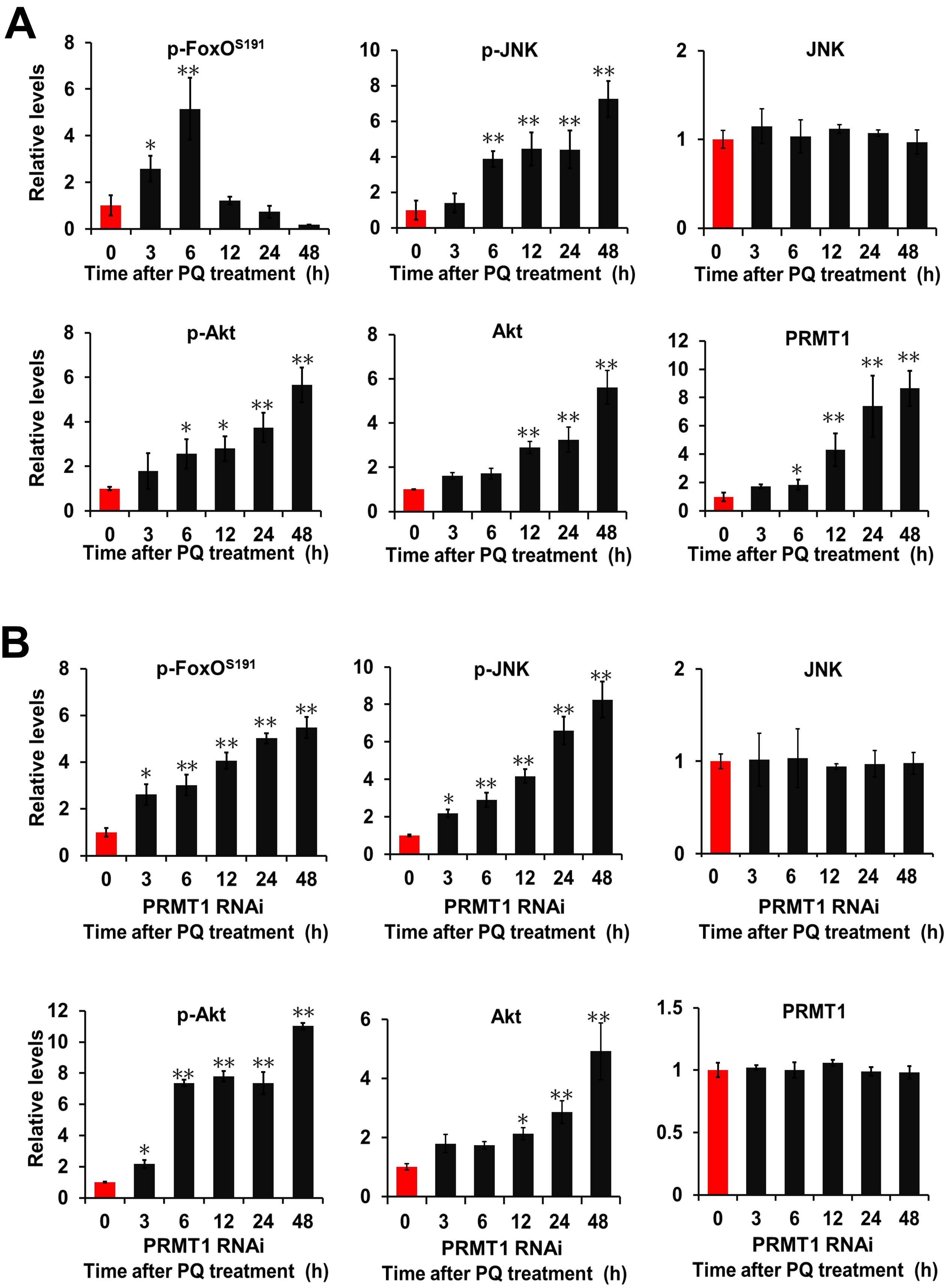
A
p-FoxO^{S191}
Relative levels
0 2 4 6 8
0 3 6 12 24 48
Time after PQ treatment (h)
p-JNK
0 2 4 6 8 10
JNK
0 1 2
p-Akt
Akt
PRMT1
0 2 4 6 8 10 12
*
**
B
PRMT1 RNAi
Time after PQ treatment (h)
p-FoxO^{S191}
p-JNK
JNK
p-Akt
Akt
PRMT1
0 0.5 1 1.5

Figure S4

Quantification of protein abundance from Figure 2. (A) Quantification of p-FoxO^{ser191}, p-JNK, JNK, p-Akt, Akt and PRMT1 from figure 2A. (B) Quantification of p-FoxO^{ser191}, p-JNK, JNK, p-Akt, Akt and PRMT1 from figure 2C. Western blot bands were quantified using Image software (Gel-Pro Analyzer) and normalized to the levels of Actin. Each point represents the mean±S.D., n=3; *, P<0.05; **, P<0.01.

Fig. S5

A

```
                                       BR-C
-1438 CGGTAATGAGTAACTAGTTTTTGTCTCTAATTGAAAATATTTCAAAAGCAAACAGATAG
                                  POU
-1378 GGTACTGCTGAAAATGAATAAAATTTATAATGCTTCGGGTCTTTACAGACTAAAAGAGATA
                                                             c-Myc
-1318 AACATCATTTTGAACTTACATTCAGGTTTTATTAGTTTATTTAAATGCAAACACGTTTTT
          CREB
-1258 GATATGAGGAAAATGAGCTCTGCAATAAATACGAAACAACATGGATATAATGTTTGTTAT
-1198 CATCTTTACAACTATTTACTCACATTATTATCTGGGCTTGCTGTTATAGCACAACTAAAC
                                        POU
-1138 CAACTCATTTTACTTTCAAATAAATGGCTTATTAAATCTTAATTTATTTTAGGTTTATGT
-1078 GTCATTTTGATAAAACATGACCACCTTGAAATCGTTTCAGTATTACCTACGTTACGTACA
-1018 TAATATAAATAGGGAGCATAGGTAGTTCAGTCACAAAATCATCCCACTATCTAGCACTTG
-958 TAACTTTATTTACCTATAGGTAGGTACAACAACAACAAAAAAACAAAAAATATTCCTCTT
-898 ATAGGTTTGCCTGGGCACAAAAAAGGCATTTCCATTCAGGTATCTTTCAGAATAAACATC
-838 GCACTATTTTACATTCATAAATTACTGTACCTAAACCACTCCTACAAATTCAGCGTTATC
-778 GTAACAAACATGTGCATTTTTACAATTTAAACTTCCATCACATATTTTACAAATAGTACC
-718 AATTTTAAAGCATCAAACGATTTTTAATAATGAATTTATTTACTTACGTACTCACGTTAC
-658 TGCTTTCGATGTCGTTATCACTTTCGATATAAAGTTTATTTCCCGCCAAAATTTACACAA
-598 ATTGACGTGCTTCTGCACCGGTATAATGTCACAAACTAAAGTGGACTCAGAATGAAGGCT
-538 GACGACCTCTGGCTTACTTTTATTTAAAAGTGATTAAATTAAAACGCTGCAGTACACGTA
-478 TTTTACCCATATGTGTGGTAAGCATGCTCGCTTTGCGAATCCATGAGTAGGAAAGAAAGT
-418 ATCAATGTTGATGATAAATAGGTCCTCAATCAAACAATAGATAAAAACATTTTGGTATTT
-358 AATTAAAAAAATTAACCTATATAAAACTACTTCACATTCAGGTTTAATGTTAGAATAATT
-298 ATTTAGAATTTATATTTAGATACAACGTCTCATCAAGAAAAGTGCAATATTGTGCGAAAT
-238 ACAATAGCCAATACCTTCAGACAATCATGCACAATATTACTTTACTCTCTGATCATGCAC
-178 TTGAATGAAGGTTAAATTGTTCAAATTTCAATGCTCTGAGTGCAGTGAAGGGCAGAAAAT
-118 GAATTAAATATCAATTGACTTTTAATATTTTGCAATTTAATAATAATCGATTTCAATCGA
-58 ATACATTTCCCAATCGAGTACAGCTATCATATTGTCCAAGCACTATCACAGTGTTTCCAG (+2)
```

B

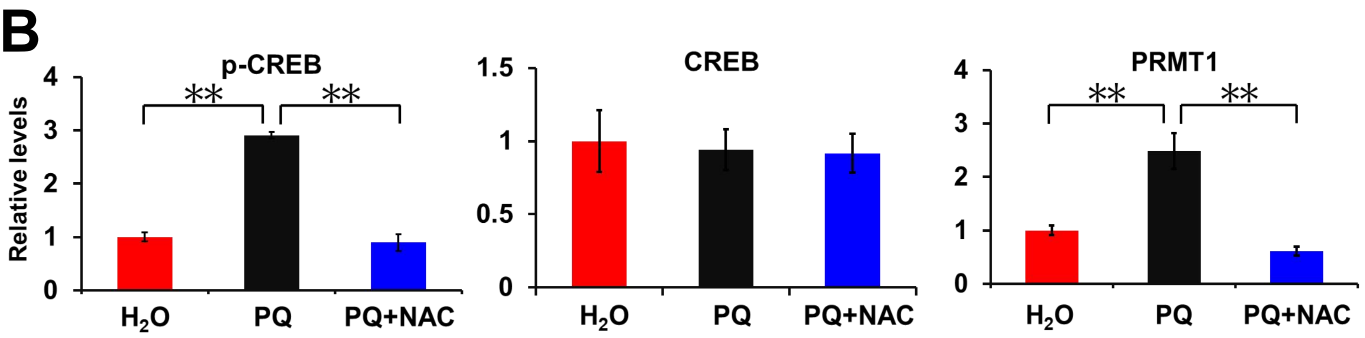

Figure S5

Nucleotide sequence of the promoter region of *H. armigera PRMT1* and quantification of protein abundance from Figure 3F.

(A) Nucleotide sequence of the *PRMT1* promoter. Nucleotide positions are numbered relative to the predicted transcription start site (indicated by the arrow). Potential consensus sequences for transcription factor-binding sites are underlined. (B) Quantification of p-CREB, CREB and PRMT1 from figure 3F. Each point represents the mean±S.D., n=3; **, P<0.01.

Fig. S6

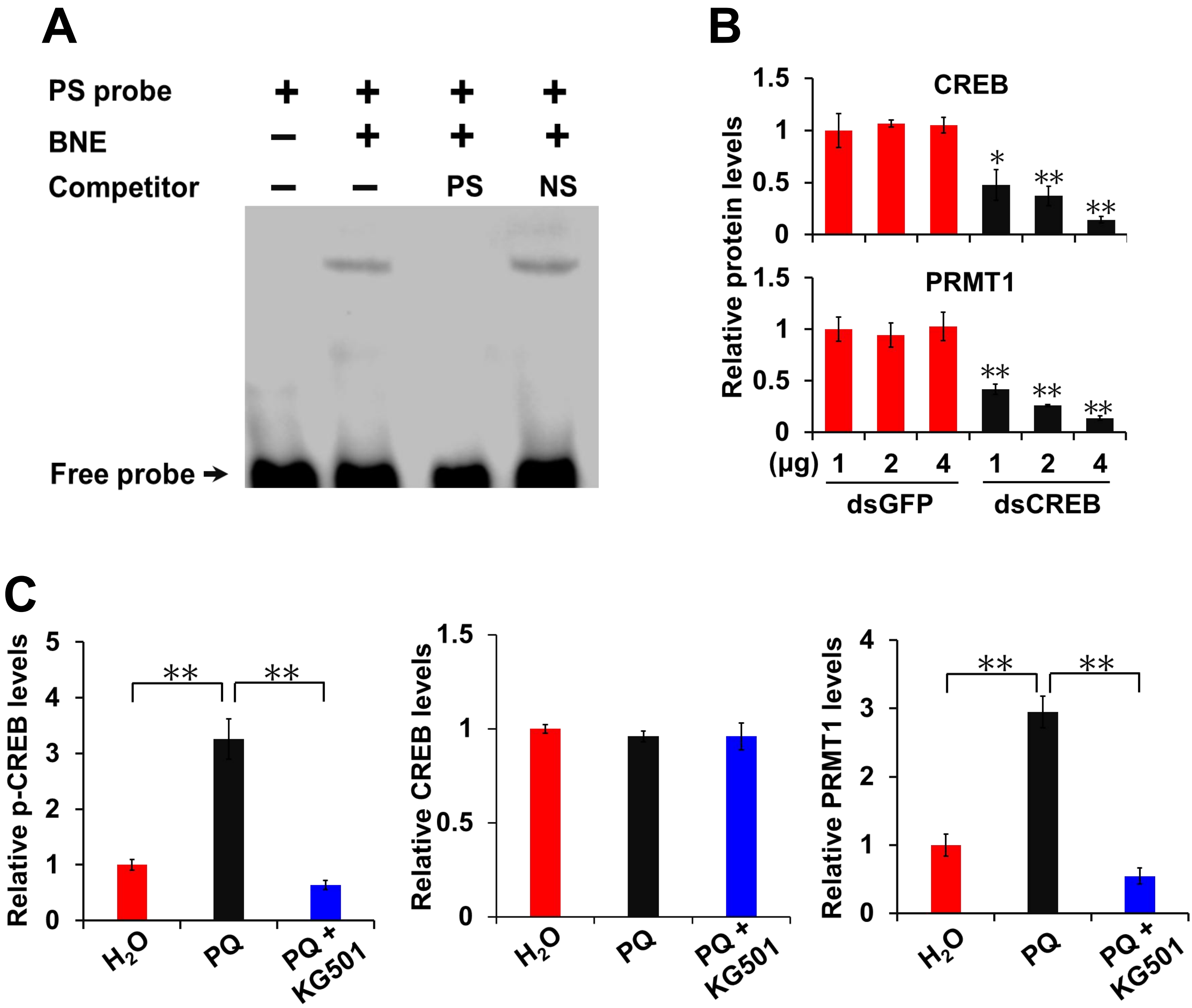
A
PS probe
BNE
Competitor
PS
NS
Free probe
B
CREB
PRMT1
Relative protein levels
(μg) 1 2 4 1 2 4
dsGFP
dsCREB
C
Relative p-CREB levels
Relative CREB levels
Relative PRMT1 levels
H2O
PQ
PQ + KG501

Figure S6

CREB binds to the *PRMT1* promoter and regulates PRMT1 expression. (A) EMSA for the CREB binding site. The probe PS was incubated with brain nuclear extract (BNE). PS, *PRMT1* promoter-specific probe; NS, non-specific competitor. (B) Quantification of CREB and PRMT1 abundance from figure 4D. (C) Quantification of p-CREB, CREB and PRMT1 abundance from figure 4E. Protein bands were quantified using Image software (Gel-Pro Analyzer) and normalized to the levels of actin. Each point represents the mean±S.D., n=3; *, P<0.05; **, P<0.01.

Fig. S7

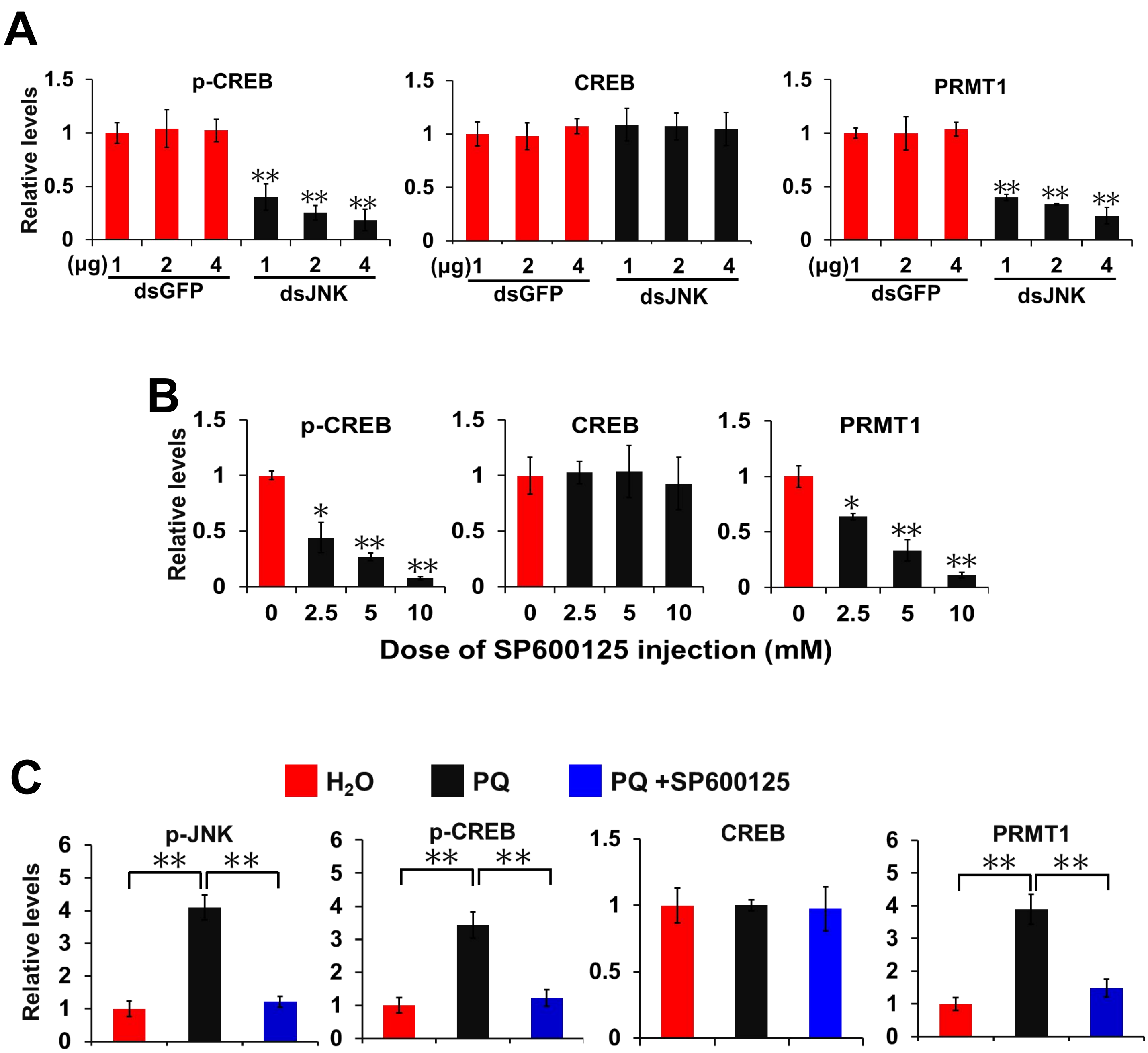
A
p-CREB
CREB
PRMT1
Relative levels
1.5
1
0.5
0
(µg) 1 2 4 1 2 4
dsGFP
dsJNK
**
B
Relative levels
Dose of SP600125 injection (mM)
0 2.5 5 10
*
C
H_2O
PQ
PQ +SP600125
p-JNK
6
5
4
3
2

Figure S7

Quantification of protein abundance from Figure 5. (A) Quantification of p-CREB, CREB and PRMT1 abundance from figure 5D. (B) Quantification of p-CREB, CREB and PRMT1 abundance from figure 5F. (C) Quantification of p-JNK, p-CREB, CREB and PRMT1 abundance from figure 5G. Protein bands were quantified using Image software (Gel-Pro Analyzer) and normalized to the levels of actin. Each point represents the mean±S.D., n=3; *, P<0.05; **, P<0.01.

Fig. S8

H. armigera FoxO	1	M S I R G S G G Y Q S P W S S Q G G H S E L D G T L E L E P L G E L T E V G F E	40
H. armigera FoxO	41	P Q T R A R S N T W P L P R P D N Y V E A A D D T G S K K N S N Q N L S G A P P	80
H. armigera FoxO	81	L P A V G T K K N S S R R N A W G N L S Y A D L I T Q A S T S A R D N R L T L S	120
H. armigera FoxO	121	Q I Y E W M I Q N V P Y F K D K G D S N S S A G W K N S I R H N L S L H N R F M	160
H. armigera FoxO	161	R V Q N E G T G K S S W W M I N P D A K P G K S V R R R A A S M E T S K F E K R	200
H. armigera FoxO	201	R G R V K K K A E I L R T G A T A D A T P S P G S S V S E S L D M F P D S P M H	240
H. armigera FoxO	241	S S F Q L S P D F R P R V S S N A S S C G R L S P I P S M I T T E H D W G P E Y	280
H. armigera FoxO	281	T D Y T S A N D Y S Q T D F G Q D E L A G S L A D S M K L A G T D P F L N T Y V	320
H. armigera FoxO	321	P T T S S S S S G G S Y R Y S P Y G G C P R H P H G G C A C S S L Y T H P T H P	360
H. armigera FoxO	361	A H P T H P H Q H A L D H F V R P P P P A D P A D I M R T V P F T E N N Q T Q M	400
H. armigera FoxO	401	V T T S D A A L M N G G M M V Q T G A M G P T T V M G R I M G A L N T G L A E D	440
H. armigera FoxO	441	L N I E T L E H G F D C N V D E V I K H E L S M E G T L D F N F P Q Q H S A M A	480
H. armigera FoxO	481	A E A E S Q F A A P A P P V P T T L S G G N G P R A P Y S V A P S W V H	516

Figure S8

Phosphorylation motif and binding domain of *H. armigera* FoxO to JNK. Phosphorylation motif and binding domain of FoxO to JNK are predicted online (http://gps.biocuckoo.cn). Red line shows JNK phosphorylation motif (PXSP), and blue line shows JNK binding domain (R/KXXXXLN/EL).

Fig. S9

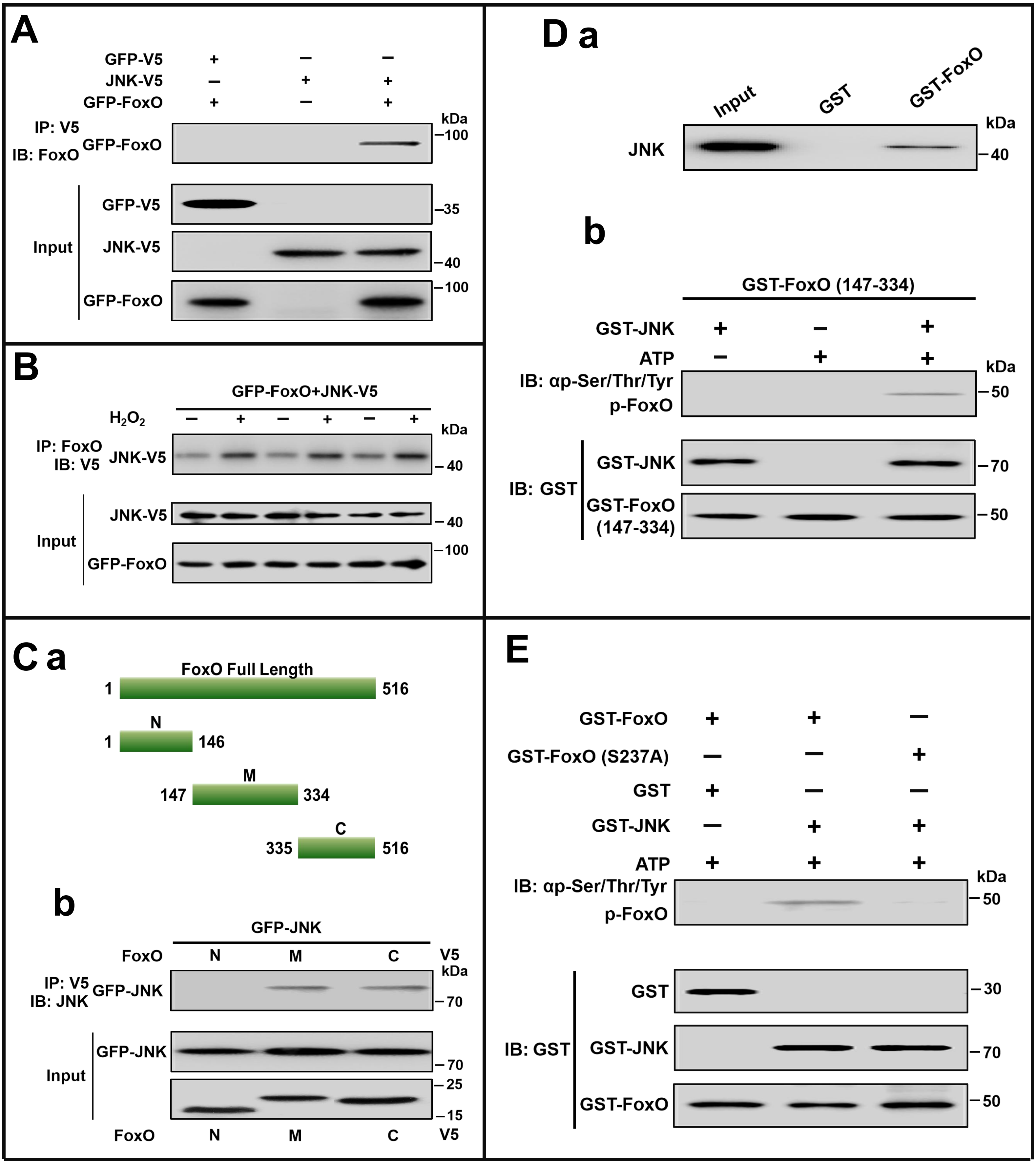
A
GFP-V5
JNK-V5
GFP-FoxO
IP: V5
IB: FoxO
GFP-FoxO
kDa
100
Input
GFP-V5
35
JNK-V5
40
GFP-FoxO
100
B
GFP-FoxO+JNK-V5
H_2O_2
IP: FoxO
IB: V5
JNK-V5
kDa
40
Input
JNK-V5
40
GFP-FoxO
100
C a
FoxO Full Length
1
516
N
1
146
M
147
334
C
335
516
b
GFP-JNK
FoxO
N
M
C
V5
kDa
IP: V5
IB: JNK
GFP-JNK
70
Input
GFP-JNK
70
25
15
FoxO
N
M
C
V5
D a
Input
GST
GST-FoxO
JNK
kDa
40
b
GST-FoxO (147-334)
GST-JNK
ATP
IB: αp-Ser/Thr/Tyr
p-FoxO
kDa
50
IB: GST
GST-JNK
70
GST-FoxO
(147-334)
50
E
GST-FoxO
GST-FoxO (S237A)
GST
GST-JNK
ATP
IB: αp-Ser/Thr/Tyr
p-FoxO
kDa
50
IB: GST
GST
30
GST-JNK
70
GST-FoxO
50

Figure S9

JNK binds to and phosphorylates FoxO and analysis of phosphorylation site of FoxO. (A) JNK physically associates with FoxO by Co-IP. HzAm1 cells were cotransfected with GFP-FoxO and JNK-V5 for 48 h, and the cell extracts were immunoprecipitated (IP) with anti-V5 antibody, followed by immunoblotting (IB) with an anti-FoxO or anti-V5 antibody, respectively. (B) Oxidative stress promotes JNK and FoxO binding. HzAm1 cells were cotransfected with GFP-FoxO and JNK-V5 for 48 h, and then were treated with 300 μM H_2O_2 for 30 min. Cell extracts were immunoprecipitated with an anti-FoxO antibody, followed by IB with an anti-V5 or anti-FoxO antibody. (C) Coimmunoprecipitation analysis of JNK with FoxO fragments. (a) Schematic representation of *H. armigera* FoxO fragments. (b) Coimmunoprecipitation analysis of JNK with FoxO fragments. Three FoxO-V5 fragments were cotransfected with GFP-JNK into HzAm1 cells for 48 h, and the cell extracts were immunoprecipitated with an anti-V5 antibody, followed by IB with an anti-JNK or anti-V5 antibody. (D) JNK directly binds to and phosphorylates FoxO. (a) JNK interacts with FoxO by GST pull-down assay. Brain extracts were incubated with purified GST or GST-FoxO, followed by IB with anti-JNK antibody. (b) *In vitro* phosphorylation assay with or without GST-JNK together with GST-FoxO in the presence or absence of ATP. Reaction products were analyzed by IB with an anti-phospho-ser/thr/tyr antibody. Total amounts of GST-FoxO and GST-JNK were assessed by IB using an anti-GST antibody. (E) *In vitro* phosphorylation assay GST-JNK together with WT, S237A mutant of GST-FoxO in the presence of ATP. GST was used as a negative control. Total amounts of GST-FoxO, GST and GST-JNK were assessed by IB using an anti-GST antibody.

Supplemental Table

List of primers used in this study

**Degenerate primers**	
JNK-F1	5'-GTYGAGGTSGGSGACAC-3'
JNK-F2	5'-CAGAACGTKACRCACGC-3'
JNK-R1	5'-GACGTAKGGMGTCATCAT-3'
JNK-R2	5'-TTGATGCCGCAMAGCAT-3'
**RACE (Rapid amplification of cDNA ends) primers for JNK**	
GSP-F	5'-CACGCCACAGAAGAGCCTGG-3'
NGSP-F	5'-GGAGTTCCAGGACGTGTACC-3'
GSP-R	5'-CAGCAGGTAGCTCATGCGC-3'
NGSP-R	5'-CGTGGTCGAGGTCCATCTG-3'
**Overexpression and ChIP primers**	
PRMT1-F	5'-CGGAATTCTATGGAGAGCATGGATGTTGCAC-3'
PRMT1-R	5'-CCCTCGAGCGGCGCATCCTATAATGATTCTTC-3'
JNK-F	5'-CGGAATTCTATGCCCCACGCGGCGCTCCCAG-3'
JNK-R	5'-CCCTCGAGCGTGTGGTGAGGGCGGGCTGCGCGTG-3'
CREB-F	5'-CGGAATTCTATGGATGGAATGGTGGAAGAG-3'
CREB-R	5'-CCCTCGAGCGTCATTCTGTCTTTTGCTGACAG-3'
FoxO-N-F	5'-CCCTCGAGCGTCATTCTGTCTTTTGCTGACAG-3'
FoxO-N-R	5'-CCGCTCGAGCGCCTCCATCCAGCCGAAGAGTTG-3'
FoxO-M-F	5'-CCGGAATTCTAACTCAATCAGACATAACCTGTC-3'
FoxO-M-R	5'-CCGCTCGAGCGGTACCGGTGGCTGCCGCCCGATG-3'
FoxO-C-F	5'-CCGGAATTCTAGCCCGTACGGCGGGTGTCCG-3'
FoxO-C-R	5'-CCCTCGAGCGGTGGACCCAGGAGGGGGCGAC-3'
PRMT1 ChIP-F	5'-GCAATTACGGTAATGAGTAACTAG-3'
PRMT1 ChIP-R	5'-GCCATTTATTTGAAAGTAAAATGAG-3'
**Oligonucleotides for EMSA**	
PS-F	5'-CGTTTTTGATATGAGGAAAATGAGCTCTGCAA-3'
PS-R	5'-TTGCAGAGCTCATTTTCCTCATATCAAAAACG-3'
MPS-F	5'-CGTTTTTGATACTCGTAACATGAGCTCTGCAA-3'
MPS-R	5'-TTGCAGAGCTCATGTTACGAGTATCAAAAACG-3'
**Primers for genome walking and stepwise deletion plasmid constructs**	
PRMT1W1	5'-ATCCACTAGCGAACTTATAAG-3'
PRMT1W2	5'-GACCAATTTAGAGTTGCCAG-3'
PP3	5'-GGGGTACCCCTATAGGTAGGTACAACAAC-3'
PP2	5'-GGGGTACCCTTACGTACTCACGTTACTGC-3'
PP1	5'-GGGGTACCACTCTCTGATCATGCACTTG-3'
Common R	5'-CGGCTAGCGACCAATTTAGAGTTGCCAG-3'
**Primers for RNAi and qPCR**	
dsJNK-F1	5'-GGATCCTAATACGACTCACTATAGGACGGAGAACGTGGACATC TGGTC-3'

dsJNK-F2	5'-ACGGAGAACGTGGACATCTGGTC-3'
dsJNK-R1	5'-GGATCCTAATACGACTCACTATAGGCGCGCTCGTCCACCGAGTGGTC-3'
dsJNK-R2	5'-CGCGCTCGTCCACCGAGTGGTC-3'
dsCREB-F1	5'-GGATCCTAATACGACTCACTATAGGGCTCCTTCTGCACAGGTCCAATC-3'
dsCREB-F2	5'-GCTCCTTCTGCACAGGTCCAATC-3'
dsCREB-R1	5'-GGATCCTAATACGACTCACTATAGGCTTCTAGCAATGGACCAGGGAC-3'
dsCREB-R2	5'-CTTCTAGCAATGGACCAGGGAC-3'
Q-PRMT1-F	5'-GCCGGAGCAGCTAAAGTCATAG-3'
Q-PRMT1-R	5'-GAACAGGCAGTAGCCCATCCAC-3'
